# Phase I study of vinblastine in combination with nilotinib in children, adolescents, and young adults with refractory or recurrent low-grade glioma

**DOI:** 10.1093/noajnl/vdaa075

**Published:** 2020-06-09

**Authors:** Stephanie Vairy, Gwénaël Le Teuff, Francisco Bautista, Emilie De Carli, Anne-Isabelle Bertozzi, Anne Pagnier, Fanny Fouyssac, Karsten Nysom, Isabelle Aerts, Pierre Leblond, Frederic Millot, Claire Berger, Sandra Canale, Angelo Paci, Vianney Poinsignon, Aurelie Chevance, Monia Ezzalfani, Dominique Vidaud, Angela Di Giannatale, Raquel Hladun-Alvaro, Francois M Petit, Gilles Vassal, Birgit Geoerger, Marie-Cécile Le Deley, Jacques Grill

**Affiliations:** 1 Department of Pediatric and Adolescent Oncology, Gustave Roussy, Villejuif, France; 2 Université Paris-Saclay, Université Paris-Sud, UVSQ, CESP, INSERM, Villejuif, France; 3 Service de Biostatistique et d’Epidémiologie, Gustave Roussy, Villejuif, France; 4 Département d’Hematologie et d’Oncologie Pediatrique, Centre Hospitalier Universitaire d’Angers, Angers, France; 5 Département d’Hematologie et d’Oncologie Pediatrique, Hopital Purpan, Centre Hospitalier Universitaire de Toulouse, Toulouse, France; 6 Département d’Hematologie et d’Oncologie Pediatrique, Centre Hospitalier Universitaire de Grenoble, La Tronche, France; 7 Département d’Hematologie et d’Oncologie Pediatrique, Centre Hospitalier Universitaire de Nancy, Nancy, France; 8 Department of Pediatric Hematology and Oncology, Rigshospitalet, Copenhagen, Denmark; 9 SIREDO Center, Institut Curie, Paris, France; 10 Unité d’oncologie pédiatrique, Centre Oscar Lambret, Lille, France; 11 Département d’Hematologie et d’Oncologie Pediatrique, Centre Hospitalier Universitaire de Poitiers, Poitiers, France; 12 Département d’Hematologie et d’Oncologie Pediatrique, Centre Hospitalier Universitaire de Saint-Etienne, Saint-Priest-en-Jarez, France; 13 University Research Team EA, SNA-EPIS, Saint-Etienne, France; 14 Department of Radiology, Gustave Roussy, Villejuif, France; 15 Department of Pharmacology and Pharmacokinetics Unit School of Pharmacy, Université Paris-Saclay, Université Paris-Sud, Gustave Roussy, Villejuif, France; 16 Service de Génétique et Biologie Moléculaires, Hopital Cochin, Hopitaux Universitaires de Paris Centre, Assistance Publique—Hôpitaux de Paris, and EA7331, Faculte de Pharmacie de Paris, Universite Paris Descartes, Paris, France; 17 Département de Génétique Moléculaire, Hopital Antoine Beclere, Clamart, France

**Keywords:** Gilbert disease, neurofibromatosis type 1, pharmacogenomics, pharmacokinetics, pilocytic astrocytoma

## Abstract

**Background:**

New rescue regimens are needed for pediatric refractory/recurrent low-grade glioma. Nilotinib is a tyrosine kinase inhibitor that has potential synergistic effects with vinblastine on angiogenesis, tumor cell growth, and immunomodulation.

**Methods:**

This phase I trial aimed to determine the recommended doses of this combination for phase II trials (RP2D) using the dual-agent Bayesian continual reassessment method. Nilotinib was given orally twice daily (BID) in combination with once-weekly vinblastine injections for a maximum of 12 cycles of 28 days (clinicaltrials.gov, NCT01884922).

**Results:**

Thirty-five pediatric patients were enrolled across 4 dose levels. The median age was 7 years and 10 had neurofibromatosis type 1. Patients had received a median of 3 prior treatment lines and 25% had received more than 4 previous treatment lines. Dose-limiting toxicity (DLT) during cycle 1 was hematologic, dermatologic, and cardiovascular. The RP2D was identified at 3 mg/m^2^ weekly for vinblastine with 230 mg/m^2^ BID for nilotinib (estimated probability of DLT = 18%; 95% credibility interval, 7–29%). Fifteen patients completed the 12 cycles; 2 stopped therapy prematurely due to toxicity and 18 due to disease progression. Three patients achieved a partial response leading to an objective response rate of 8.8% (95% confidence interval [CI], 1.9–23.7), and the disease control rate was 85.3% (95% CI, 68.9–95.1). The 12-month progression-free survival was 37.1% (95% CI, 23.2–53.67).

**Conclusions:**

Vinblastine and nilotinib combination was mostly limited by myelosuppression and dermatologic toxicity. The efficacy of the combination at the RP2D is currently evaluated in a randomized phase II trial comparing this regimen to vinblastine alone.

**Key Points**

- Vinblastine and nilotinib combination is well tolerated.
- The main toxicities associated with this combination are hematologic and dermatologic.
- This regimen will be further evaluated in a phase II study against vinblastine monotherapy.

**Importance of the Study**

Chemotherapy is the mainstay for the treatment of the unresectable progressive low-grade gliomas in children. More effective and less toxic regimens need to be explored in this population. Here, we establish the recommended phase II dose of vinblastine, a known effective chemotherapy for this kind of neoplasm, combined with nilotinib, a tyrosine kinase inhibitor, applying a dual-agent Bayesian continual reassessment method for dose escalation. Thirty-five patients, the majority having optic pathway gliomas, were treated after multiple previous lines of chemotherapy. Dose-limiting toxicities were mostly hematologic and dermatologic and were manageable. Despite the impossibility to escalate the dose of vinblastine above 3 mg/m^2^ weekly, long-lasting responses were observed and justify the comparison of this combination against vinblastine monotherapy in a randomized clinical phase II trial.

Low-grade gliomas (LGGs) represent 30–40% of central nervous system (CNS) tumors in children, with an incidence of 10–12 per 1 000 000 children younger than 15 years in Western countries.^1,2^

Surgery is the treatment of choice but new therapeutic options are needed for unresectable tumors and for children suffering recurrences. Chemotherapy was shown to delay or avoid radiation therapy and so, potentially serious long-term sequelae. Indeed, in some of these patients, objective response rate of 42–85% and radiotherapy-free survival rate of about 61–83% are described.^3–5^ Due to multiple relapses/progressions, patients with LGG may need more than one regimen to control the disease while protecting the child’s brain from the neurological and cognitive deficits associated with both the tumor itself and aggressive multimodal therapy.^6–8^ It is therefore desirable to develop effective drug combinations that can be safely administered during a prolonged period.

Some publications highlight the prognostic role of neovascularization, and the risk for progression has been significantly associated with a high micro-vascularization density (>20 vessels/mm^2^).^9^ Vinblastine vascular cytotoxicity is thought to have an indirect cytotoxic effect on tumors, because their growth is dependent on angiogenesis. It has in fact been demonstrated that vinblastine causes patchy necrosis of tumor cells closely associated with cytotoxic damage and necrosis of the vascular endothelial cells in animals.^10,11^

Platelet-derived growth factors (PDGFs) are known as growth factors for normal and tumoral astrocytes and oligodendrocytes.^12–14^ PDGF receptor alpha and beta (PDGFR-α and PDGFR-β) are found to be overexpressed in LGGs, and PDGFR-β is particularly expressed in the developing vasculature, including tumor angiogenesis.^15–18^

LGGs are associated with a mixture of inflammatory and immune cells and, particularly for pilocytic astrocytoma, a macrophage population is dominant peri-vascularly.^19^ Gutmann et al.^20–22^ also reported that in neurofibromatosis 1 (NF1)-associated LGG, microglia were present in increased number and facilitated glial proliferation in the early phase of optic pathway glioma development. Interestingly, microglia also seems to induce PDGFR-β expression in glioma cells, enhancing the migratory and invasive capacity of glioma cells.^23^ Various chemotherapies display immuno-stimulatory properties. Among them, vinblastine may enhance the anticancer immune response by dendritic cells (DCs) maturation.^24^ By inhibiting stem cell factor receptor (KIT) in DCs, imatinib can promote a DC/natural killer (NK) crosstalk that ultimately stimulates NK cells to produce IFN-γ both in mice and in humans.^25^ Nilotinib also displays immunomodulation and anti-inflammatory properties that could be of interest to induce tumoral stroma modifications, which has been considered as a key in the development of these tumors.^26^

Imatinib improves the intratumoral bioavailability of chemotherapeutic approaches diminishing interstitial pressure suggesting its use in combination with chemotherapy.^27,28^ Clinical tumor response to the c-KIT/PDGFR/ABL tyrosine kinase inhibitor (TKI) imatinib has been occasionally reported in LGG.^15,29,30^ Nilotinib is a second-generation TKI developed as a selective and potent inhibitor of the TK activity of BCR-ABL. Similar to imatinib, nilotinib inhibits the TK activity of the PDGFR-α and β, KIT, colony-stimulating factor receptor (CSF-1R), discoidin domain receptor (DDR), and ephrin-A4 receptor (EPHA4) kinases and blocks the downstream cellular events mediated by these enzymes.^31^ A comparison of imatinib and nilotinib for effects on autophosphorylation and proliferation in cells expressing PDGFR-α/β showed nilotinib to be more potent than imatinib.^32^ While few data exist about CNS penetrance of nilotinib, one study reported improved clinical efficacy over imatinib in a small series of patients with meningeal relapse of leukemia. Despite the low cerebrospinal fluid (CSF)/plasma ratio observed, this may be explained by a higher amount of free and active nilotinib in the CSF.^33^ Pediatric clinical experience with nilotinib has been mainly described in Philadelphia-positive leukemia resistant or intolerant to imatinib or dasatinib and toxicity profile has been established in this population.^34^

Taking advantage of their different antiangiogenic and antitumoral mechanisms described above, their limited and nonoverlapping toxicities, vinblastine and nilotinib could play an interesting role in the treatment of pediatric LGGs. We here report the result of the phase I dose-finding study of the VINILO trial (NCT01884922) which is an open-label, international trial evaluating the combination of nilotinib with vinblastine in young patients with recurrent/refractory LGG.

## Methods

### Study Population

Patients enrolled in this phase I trial were from France and Denmark. Eligibility criteria included age between 6 months and younger than 21 years, refractory or recurrent LGG after at least one first-line therapy, Lansky play scale or Karnofsky performance status at least 70%, life expectancy at least 3 months, and adequate organ functions. Histological documentation was mandatory in non-NF1 patients. Refractory disease was defined as radiographic or clinical progressive disease while on treatment. If steroids were administered, the dose had to be stable for 1 week and patients should not have peripheral neuropathy of at least grade 2 (NCI—common terminology criteria for adverse event CTC AE v4.0). Exclusion criteria were uncontrolled infection, severe systemic disease, gastrointestinal disease (which could lead to malabsorption of nilotinib), grade 2 and above toxicity from prior treatment, known intolerance or hypersensitivity to vinblastine, and simultaneous treatment with strong cytochromes P450 CYP3A4 inhibitors or drugs known to prolong QT interval. Previous treatment with one of the study drugs was not an exclusion criterion. The study received regulatory approvals from a national drug security agency, institutional review boards, and ethical approval in each country and center. Patients and/or their legal guardians gave written informed consent, and assent was obtained as appropriate at the time of enrollment.

### Trial Design and Treatment

The phase I was an open-label, non-randomized, sequential dose escalation of vinblastine and nilotinib. The objective was to identify the recommended dose of the combination of vinblastine and nilotinib associated with an estimated probability of dose-limiting toxicity (DLT) deemed acceptable, evaluated on the first 28-day treatment cycle. Vinblastine was administered in a 15-min infusion once a week. Nilotinib was given orally twice daily (BID), continuously. The planned evaluated doses were 3, 4, 5, and 6 mg/m^2^ for weekly vinblastine and 115, 230, and 350 mg/m^2^ for nilotinib BID, with dose rounded to the nearest 50 mg. There was no intra-patient dose escalation. For safety reasons, the starting dose level of 3 mg/m^2^ for vinblastine combined with 115 mg/m^2^ for nilotinib, hereinafter denoted (3;115), corresponded to 50% of the recommended dose of each agent given separately. Dose allocation was centrally defined, based on toxicity observed in patients previously evaluated, by modeling the probability of DLT (see definition in the Safety Evaluation section). During the dose escalation, every new patient was treated at the best current recommended dose, ie, the dose associated with an estimated probability of toxicity deemed acceptable (target toxicity probability set at 20%) for this patient population. Indeed, the choice of a conservative 20% as target toxicity rate was based on the need for a tolerable chronic treatment in this specific pediatric population with frequent relapses.

Based on a simulation study detailed in Supplementary Data II, the dual-dimensional Bayesian continual reassessment method (CRM) published by Wang and Ivanova^35^ was applied, which is an extension of the CRM published by O’Quigley et al.^36^ We assumed there was no interaction between the 2 agents and no start-up (ie, no accumulation of data not guided by the model). We used a slightly modified CRM design, avoiding waiting lists when the toxicity assessment of the patient(s) previously recruited was not complete.^37^ At least 2 patients with a complete evaluation within the first cycle and with no DLT were requested at a given dose level before dose escalation. No skipping dose was allowed.

We first aimed to escalate the nilotinib dose from 115 to 230 mg/m^2^ BID with a stable vinblastine dose of 3 mg/m^2^, dose levels (3;115) and (3;230), respectively. As soon as the current dose level was deemed safe, it was initially planned to escalate the dose of vinblastine to 4, 5, and 6 mg/m^2^ with a stable dose of nilotinib 230 mg/m^2^ BID, (4;230), (5;230), and (6;230), respectively. In the initial version of the protocol, nilotinib 350 mg/m^2^ was to be explored only if the dose level (5;230) was deemed safe. However, after the protocol amendment, we decided to also explore the combination of nilotinib 350 mg/m^2^ BID with vinblastine 3 and 4 mg/m^2^, (3;350) and (4;350), respectively. The decision to explore the different possible adjacent doses was based on the posterior probability of DLT, estimated by the model for all the dose combinations at the time of each new inclusion.

Patients who had failed to complete the first cycle of treatment or who received a reduced dose of study drugs (<70% of the planned dose of vinblastine or <80% of the planned dose of nilotinib) because of protocol violation or for a reason other than study drug-related adverse event were considered as not evaluable for the dose-escalation phase.

Treatment was continued until disease progression, unacceptable toxicity, inadequate toxicity–benefit ratio, or patient/parents decision to no longer participate in the study. The maximum treatment duration was 12 cycles.

### Safety Evaluation

Safety of the study treatment was evaluated based on the clinical and biological evaluation, including a complete blood count once a week during the first 3 cycles, then at least before each cycle during the study treatment period and biochemistry tests before each 28-day cycle (details in Supplementary Data I).

The dose-finding part of the trial was driven by the occurrence of DLTs (primary endpoint), assessed over the first 28-day cycle. We defined hematological DLT as any grade ≥3 neutropenia (<1 × 10^9^/L) for more than 7 days and grade ≥2 thrombocytopenia (<75 × 10^9^/L) or thrombocytopenia requiring transfusions for more than 7 days. We also defined DLT as any grade ≥3 nonhematological toxicity, excluding grade 3 nausea, vomiting, fever, rapidly reversible hepatic toxicity (ie, returns to <2.5 × upper limit of normal (ULN) within 2 weeks after study drug discontinuation), as well as symptoms unequivocally related to tumor progression. Severity of adverse events was graded according to NCI-CTCAE version 4.0. In addition, we also considered as a DLT any study drug-related toxicity leading to a significant dose reduction over the first cycle, considering the first cycle plus the following week (first week of cycle 2), ie, if the patient received less than 70% of the planned dose of vinblastine (omission of more than 1 injection) or less than 80% of the planned dose of nilotinib.

Adverse events classified as related to study treatment or not were also reported over the whole treatment duration, except adverse events unequivocally related to the underlying disease or its progression.

Serious adverse events (SAEs) were defined as any untoward medical occurrence, at any dose, that was life-threatening, resulted in death, persistent or significant disability, birth defect, required (or extended) hospitalization, or considered medically significant.

### Tumor Evaluations

Every 3 cycles, tumor assessment was done by MRI with at least 2 plans of gadolinium-enhanced T1 sequences (sagittal, axial, and/or coronal) mandatory, with T2 and FLAIR sequences. Progression-free survival (PFS) is the common clinical criteria adopted for analyzing the efficacy of a chemotherapeutic approach in LGGs. Ophtalmologic status was assessed whenever possible if the patient had chiasmatic/optic pathway glioma (visual acuity by Snellen chart or equivalent, ocular fundus, and Goldmann or computer perimetry visual field). We considered clinical progression such as vision deterioration without radiological progression as an event. Objective response (minor, partial, and complete responses) was defined on the basis of the radiological tumor responses according to RANO criteria for LGG specifically, and disease control rate (DCR) included objective responses and stable disease.^38^ Imaging was centrally reviewed at the coordinating institution. Overall survival (OS) was defined as the time from the start of study treatment to the date of death whatever the cause. Patients with no event were censored at the date of the last follow-up visit. These endpoints (OS and PFS) were only exploratory in the phase I of this study.

### Pharmacokinetics Studies

As the pharmacokinetics (PK) of nilotinib was described in patients with leukemia and interaction with vinblastine was not expected, pharmacokinetic sampling was carried out according to a limited sampling methodology. Thus, PK sampling for nilotinib was performed depending on clinical context and patient’s toxicity to determine associated trough concentration (*C*_trough_). Nilotinib concentrations were determined in 0.5 mL of plasma. Therefore, 2.5 mL of whole blood per sample were taken, centrifuged at 4000 rpm for 5 min, and the plasma frozen at −20°C. Analyses were conducted by liquid chromatography coupled to a tandem mass spectrometry after liquid–liquid extraction from plasma samples.

### Pharmacogenetics Studies

Gilbert disease analysis was done in a restricted sample of patients. For *UGT1A1* gene promoter polymorphism analysis, DNA was extracted from 5 mL EDTA blood. Numerical variations in dinucleotide repeats in the *UGT1A1* (TA(n)TAA) promoter were genotyped by length measure analysis on an ABI PRISM 3130 (Applied Biosystems). Patients were considered heterozygous if (TA)6/(TA)7 or 6/7 and homozygous if (TA)7/(TA)7 or 7/7.

### Statistical Considerations

Considering the wide range of dose combinations that could be explored, we planned to recruit between 12 and 50 patients in this dose-finding part of the VINILO trial. Descriptive statistics were used to characterize the study population, safety data, and administered treatments. For each toxicity type, toxicity was reported per 28-day cycle considering the highest toxicity grade, as well as over the whole treatment duration. For treatment description, the dose intensity of each drug administered was computed, over the first cycle of treatment and over the whole treatment duration. Based on all available patients at the end of the trial, we estimated the posterior probability of DLT with its 95% credibility interval (95% CrI) for each explored dose level combination, using the highest posterior density function. PFS and OS were estimated using the Kaplan–Meier method, on all patients who started treatment, regardless of dose level. Responses (objective response rate [partial + complete response], ORR and disease control rate [all responses and stable disease], DCR) and survival data (OS and PFS) were updated in April 2019. Efficacy outcomes were estimated with their 95% confidence intervals (95% CIs).

## Results

### Patients’ Characteristics

From July 2013 to July 2015, 35 patients were included in this phase I study. The median time from initial diagnosis (date of biopsy or initial surgery) to registration was 5.1 years (interquartile range: 3.5–7.9). Patient’s age at study entry ranged from 1 to 19 years, with a median of 7 years (interquartile range: 5–10). Females accounted for 63% of the patients. Patient and disease characteristics are summarized in Table 1.

**Table 1:** Patient and Disease Characteristics at Study Entry

Characteristics	*N* = 35 (%)
Gender	
Male	13 (37%)
Female	22 (63%)
Age	
Median (range)	7 (1–19)
Q1–Q3	5–10
Neurofibromatosis type 1^a^	10 (29%)
Histological diagnosis (27 samples available)	
Pilocytic astrocytoma (WHO grade I)	19 (69%)
Astrocytoma (WHO grade II)	4 (15%)
Ganglioglioma	1 (4%)
LGG NOS	3 (12%)
BRAF rearrangement (16/27 samples available)	12
BRAF V600E mutation (21/27 samples available)	0
Primary tumor site	
Hemispheric (frontal + temporal + parietal)	1 (3%)
Cerebellum	2 (6%)
Brain stem	3 (9%)
Optic Pathway (OPG), including (possibly associated)	28 (80%)
Retrobulbar-prechiasmatic lesions	7
Chiasmatic lesions	13
Optic tract lesions	4
Hypothalamic lesions	13
No specific primary site—metastatic disease^b^	1 (3%)
Visual acuity, for the 29 patients with OPG	
No major impairment	6 (21%)
Major impairment (<3/10) in one eye	6 (21%)
Major impairment (<3/10) in both eyes	12 (41%)
Abnormal vision, NOS	4 (14%)
Missing information	1 (4%)
Metastases at study entry	2 (6%)
Type of prior lines of treatment	
Systemic therapy (chemotherapy, targeted agents)	35 (100%)
Radiation therapy	2 (6%)
Surgery	22 (63%)
Number of prior lines of treatment	
Median (range)	3 (1–10)
1	8 (23%)
2	9 (26%)
3	9 (26%)
≥4	9 (26%)
Best tumor response over the whole prior treatments	
Partial response	25 (71%)
Stable disease	7 (20%)
Progression	3 (9%)
PFS1 (in months, after the last line of treatment)^c^	
Median (range)	23.2 (1.3–148.6)
Previous systemic cancer therapy	
Vincristine	35 (100%)
Carboplatin	34 (97%)
Cyclophosphamide	22 (63%)
Cisplatin	21 (60%)
Bevacizumab	20 (57%)
Irinotecan	18 (51%)
Vinblastine	18 (51%)
Etoposide	14 (40%)
Procarbazine	11 (31%)
Others^d^	11 (31%)

LGG, low-grade glioma; NOS, not otherwise specified.

^a^Excluding one patient with NF1 mosaicism.

^b^One patient with optic pathway, brain stem, cerebellum, and medulla.

^c^PFS1: progression-free survival time observed from the most recent prior anticancer treatment.

^d^Others: thioguanine *n* = 2, thalidomide *n* = 1, temozolomide *n* = 2, methotrexate *n* = 1, hydroxyurea *n* = 1, fluvastatin *n* = 1, celecoxib *n* = 1, and CCNU *n* = 2.

Considering the prior lines of treatment, all had received prior chemotherapy, including vinblastine in 18 cases (51%), but none had received nilotinib. The carboplatin–vincristine combination was the most frequent protocol used as first-line treatment (19 patients), 2 had prior radiation therapy, and 5 patients were treated with surgery alone as a first-line treatment.

### Treatment Exposure and Safety

#### DLT assessment

All the patients started treatment with the allocated dose ±10% for both drugs. Five patients were classified as nonevaluable for DLT because of a major dose reduction or an early stop of treatment not due to study drug-related toxicity (1 Lyme disease, 2 protocol violations, 1 progressive disease, and 1 patient undergoing surgery). Overall, 9 of 30 patients evaluable for DLT had a major dose reduction as per definition.

As described above and detailed in Supplementary Data III—Table S10, the CRM design allowed to escalate, de-escalate, and re-escalate depending on DLTs occurring in previous patients, to target a DLT probability of 20%. Dose level (3;230) was opened to recruitment after 2 patients were treated at dose level (3;115), with no DLT. The dose was de-escalated after a DLT occurred at dose level (3;230), but it could then be re-escalated, and higher dose levels were explored, ie, (4;230) and (3;350). However, these 2 latter dose levels, adjacent to (3;230), were found insufficiently safe compared to the dose level (3;230). Of note, a frequentist probability calculation of 20% could have allowed us to further explore the dose level (3;350). However, we used the Bayesian method and stayed on the conservative side given our specific pediatric population, targeting the lowest toxicity possible at this point. As 17 evaluable patients had already been treated at this dose level, the dose escalation was thus stopped.

Overall, 6 patients experienced a DLT as detailed in Table 2: 2 DLTs/17 evaluable patients at dose (3;230); 3 DLTs/5 evaluable patients at dose (4;230); and 1 DLT/5 evaluable patients at dose level (3;350).

**Table 2:** Description of Dose-Limiting Toxicities (DLTs)

Patient study number - Dose level	Type of DLT
6—(3;230)	Grade 3 maculo-papular rash leading to a significant dose reduction of nilotinib
14—(4;230)	Grade ≥3 neutropenia (<1 × 10^9^/L) for more than 7 days (grade 3 at week 3 and grade 4 at week 4 of cycle 1) leading to a significant dose reduction of vinblastine and nilotinib
15—(4;230)	Grade ≥3 neutropenia (<1 × 10^9^/L) for more than 7 days (grade 3 at week 3 and week 4 of cycle 1) leading to a significant dose reduction of vinblastine
24—(3;230)	Grade ≥3 neutropenia (<1 × 10^9^/L) for more than 7 days (grade 3 at week 3 and grade 4 at week 4 of cycle 1) leading to a significant dose reduction of vinblastine
32—(4;230)	Grade 4 hypertension with seizure associated with febrile neutropenia leading to the definitive stop of treatment
34—(3;350)	Dose reduction of nilotinib related to grade 2 dermatological toxicity

Based on all evaluable patients during the first 28-days cycle (*n* = 30), the dose level of 3 mg/m^2^ weekly of vinblastine combined with 230 mg/m^2^ BID of nilotinib was associated with a predicted probability of toxicity of 0.18 (95% CrI, 0.07–0.29) (Table 3). As it represents the dosage associated with a DLT probability closest to the target of 0.20, it was thus defined as the recommended phase II dose (RP2D).

**Table 3:** Summary of Doses Allocation, Observed Dose-Limiting Toxicities (DLTs), and Estimated Posterior Toxicity Probability Per Dose Level

	Vinblastine 3 mg/m^2^	Vinblastine 4 mg/m^2^	Vinblastine 5 mg/m^2^	Vinblastine 6 mg/m^2^
Nilotinib 350 mg/m^2^	(3;350) 1 DLT/5 patients P(DLT) = 0.22 (95% CrI, 0.08–0.36)	Not explored, because of safety issue	Not explored, because of safety issue	Not explored, because of safety issue
Nilotinib 230 mg/m^2^	(3;230) 2 DLT/17 patients (+3 nonevaluable) P(DLT) = 0.18 (95% CrI, 0.07–0.29)	(4;230) 3 DLT/5 patients P(DLT) = 0.24 (95% CrI, 0.11–0.40)	Not explored, because of safety issue	Not explored, because of safety issue
Nilotinib 115 mg/m^2^	(3;115) 0 DLT/3 patients (+2 nonevaluable) P(DLT) = 0.13 (95% CrI, 0.05–0.23)	Not explored, per design	Not explored, per design	Not explored, per design

P(DLT), posterior probability of DLT, based on all observations; 95% CrI, 95% credibility interval.

#### Overall toxicity assessment

The overall treatment exposure was deemed safe and feasible. A total of 246 cycles were administered in the 35 patients with a median number of cycles of 5 (range: 1–12). Two patients received only 1 cycle. Fourteen patients (40%) completed the 12 cycles of treatment. Regarding the 21 patients who received less than 12 cycles, 2 patients stopped study treatment because of adverse events (1 patient treated at dose level (4;230) with grade 4 hypertension and 1 patient at (3;350) with grade 3 febrile neutropenia) and 18 patients due to progressive disease. Overall, 12 patients (34%) had a major modification of treatment (significant dose reduction or stop of treatment due to toxicity), including 6 of the 20 patients (30%) treated at the recommended dose (3;230). Toxicity over the whole treatment duration was deemed acceptable. As detailed in Table 4, and Supplementary Data IV—Tables S11 and S12, neutropenia was the most frequent adverse event of grade 3 or more as 17 of 35 patients (49%) experienced at least one episode of grade 3 or 4 neutropenia, including 12 patients who experienced repeated episodes of neutropenia (maximum 6 cycles with neutropenia). However, only 2 patients experienced febrile neutropenia (1 and 3 episodes, respectively). Apart from hepatic enzyme increase, other adverse events of grade 3 or higher were rare, each of them occurring in only 1 or 2 patients. No increased QTc was observed during the study.

**Table 4:** Number of Patients With NCI-CTCAE Grade ≥3 Adverse Events (AEs) and Number of Serious Adverse Events (SAEs), Overall and by Dose Level

Dose level	(3;115) *n* = 5		(3;230) *n* = 20		(4;230) *n* = 5		(3;350) n = 5		Overall	
Type of adverse event	*n*	%	*n*	%	*n*	%	*n*	%	*n*	%
Any type of adverse event	3	60	13	65	5	100	5	100	26	74
Blood and lymphatic system disorders, any type	3	60	7	35	5	100	3	60	18	51
Anemia	0		0		1	20	0		1	3
Febrile neutropenia	0		0		1	20	1	20	2	6
Neutrophil count decreased	3	60	7	35	4	80	3	60	17	49
White blood cell decreased	1	20	1	5	2	40	1	20	5	14
Gastrointestinal disorders, any type	0		1	5	0		0		1	3
Vomiting	0		1	5	0		0		1	3
General disorders, any type	0		1	5	0		0		1	3
Fever	0		1	5	0		0		1	3
Infections and infestations, any type	0		1	5	0		0		1	3
Upper respiratory infection	0		1	5	0		0		1	3
Investigations, any type	0		5	25	1	20	4	80	10	29
ALT increased	0		3	15	0		2	40	5	14
AST increased	0		1	5	0		1	20	2	6
Blood bilirubin increased	0		0		1	20	0		1	3
Hyperkalemia	0		0		0		1	20	1	3
Hypocalcemia	0		1	5	0		0		1	3
Hypokalemia	0		1	5	0		0		1	3
Hyponatremia	0		1	5	0		1	20	2	6
Lipase increased	0		0		0		1	20	1	3
Nervous system disorders, any type	0		1	5	0		0		1	3
Seizure	0		1	5	0		0		1	3
Skin disorders, any type	0		1	5	0		0		1	3
Rash	0		1	5	0		0		1	3
Vascular disorders, any type	0		0		1	20	0		1	3
Hypertension	0		0		1	20	0		1	3
SAE, any type	0		4	20	1	20	1	20	6	17

ALT, alanine aminotransferase; AST, aspartate aminotransferase.

The adverse event terms have been coded according to MedDRA classification, by System Organ Class (SOC). From a clinical perspective, we have considered all hematological AEs in the SOC “Blood and lymphatic system disorders.” We have also pooled in the SOC “Investigation” all biological AEs other than hematological AE.

For each type of adverse event, we considered the maximum grade over the whole treatment duration. We only excluded adverse events unequivocally related to the disease.

Numbers of patients with an SAE are reported for all SAE whatever the NCI-CTCAE grading.

The percentages equal to 0 have been removed from the table to facilitate the reading.

A total of 6 patients experienced at least one SAE. All the SAEs were considered as expected and were amenable to corrective therapy and reversible. One patient treated at dose level (4;230) experienced a grade 4 hypertension emergency accompanied with seizures, as well as grade 3 concomitant febrile neutropenia at day 13 of cycle 1.

### Pharmacokinetics Studies

Dosage of nilotinib plasma concentrations was assessed in 9 patients (18 samples) who experienced clinical toxicity or drug dosing modifications as summarized in Supplementary Data V—Table S13. Trough concentration (*C*_trough_) at steady state was mainly measured between 500 and 1500 ng/mL in our sample of patients.

One patient with NF1 received a third of the initial dose as an adaptation to toxicity and was still in the therapeutic level (mean *C*_trough_: 923.67 ng/mL) and had no *UGT1A1* polymorphism. The patient who had experienced grade 4 hypertension had a drug dosage 4 days after the event and discontinuation of study drug. Nilotinib was still at a significant level (123 ng/mL) and remained detectable after 11 days (6.2 ng/mL); an accidental drug overdosing was excluded.

### Pharmacogenetic of UGT1A1 Promoter Polymorphism

Due to indirect hyperbilirubinemia and myelosuppression observed in several patients and the known metabolism by cytochromes of nilotinib, we performed additional pharmacogenetic assessments. A total of 15 patients had *UGT1A1* promoter polymorphism analysis done. Eight patients (57.1%) were heterozygous (6/7) and 2 (14.4%) homozygous for a longer version of the TATAA sequence (7/7). Both homozygous patients developed indirect hyperbilirubinemia and jaundice after nilotinib exposure.

One patient with homozygous genotype developed grade 2 hyperbilirubinemia and grade 2 skin toxicity on day 7 after the start of nilotinib. Treatment was stopped for 1 week and bilirubin and skin toxicity improved to grade 1. Nilotinib was resumed at half of the dose intermittently depending on the bilirubin level and to full doses afterward. This patient also had grade 3 neutropenia during cycles 2 and 3.

Among the 8 heterozygous patients, 5 also presented neutropenia. Patient 15 presented the highest bilirubin value (5.03 mg/dL, grade 3) after the start of treatment (day 35); he had a (TA)6/(TA)7 genotype. Nilotinib was interrupted at that time when he also developed a grade 3 neutropenia. Bilirubin concentration became grade 1 a week later and nilotinib was restarted at previous doses. The trough level at day 21/cycle 8 was 1523 ng/mL (see Supplementary Data V—Table S13). The 5 coding exons of the *UGT1A1* gene were amplified and sequenced, bilirubin spectrometry was performed, but no other abnormalities were identified that could explain this value.

Patients who were either heterozygous (6/7) or homozygous (7/7) had higher maximum mean bilirubin concentration (1.84 mg/dL, ± 1.25) than those with the wild-type genotype (0.98 mg/dL, ±0.2; *P* = .2). Only heterozygous or homozygous patients developed grade 2 or higher bilirubin toxicity compared to the wild-type population, although this difference was not statistically significant (*P* = .08).

### Efficacy

**Figure 1.**
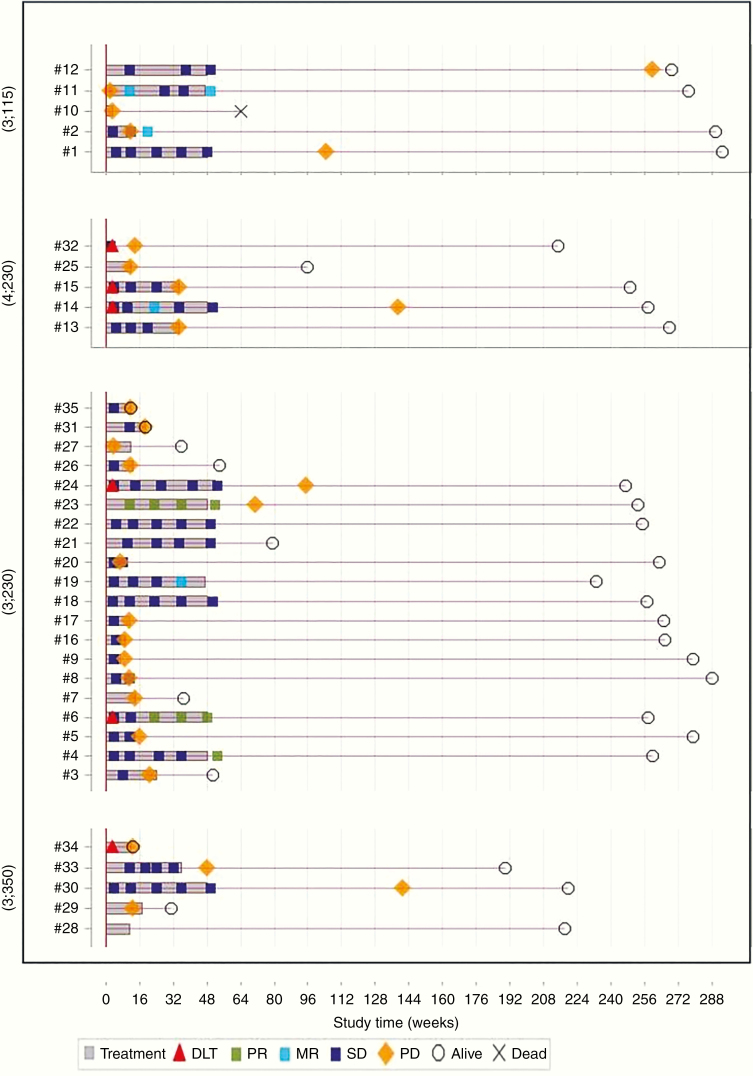
Individual description of the course of treatment for the 35 enrolled patients according to the dose level of nilotinib and vinblastine. DLT, dose-limiting toxicity; MR, minor response; SD, stable disease; PD, progressive disease; PR, partial response.

Overall, among the 34 patients classified as evaluable for best response evaluation at any time after the treatment initiated (all patients but one who stopped treatment during the first cycle due to hypertension), 3 patients achieved a partial response (>50% reduction) and 4 a minor response (>25% reduction); disease stabilization was obtained in 22 whereas 5 patients had progressive disease (including one patient with visual deterioration without radiologic modification). This leads to an ORR of 8.8% (95% CI, 1.9–23.7) and a DCR of 85.3% (95% CI, 68.9–95.1). Individual patient history over the whole study period is displayed in Figure 1. Among patients with NF1 (*n* = 10), 2 had partial response and 7 had stable disease.

We performed a sensitivity analysis considering patients treated at the recommended dose for at least 3 cycles at at least 50% of the planned doses (*n* = 18): 2 experienced partial response (11.1%), 1 had minor response (5.6%), 13 had disease stabilization (72.2%), and 2 had progressive disease (11.1%).

In the subgroup of 18 patients who had previously received vinblastine, 3 (16.7%) had minor response, 14 (77.8%) had stable disease, and 1 (5.6%) had progressive disease.

Ophthalmologic evaluations at baseline and at the end of treatment (EOT) were available for 22 patients. Among them, 7 presented deterioration in their visual evaluation, 13 were stable at the EOT with vinblastine and nilotinib, and 2 patients have their vision slightly improved. Of note, 1 patient presented a late response (stable disease in FLAIR with the disappearance of gadolinium enhancement) 6 months after treatment despite radiologic progressive disease but stable visual evaluation at EOT. Regarding the 2 patients with visual improvement, 1 presented a partial response at the EOT and a stable disease thereafter, while the other one had stable disease but relapsed shortly after the EOT. Among the patients with visual deterioration, 2 had radiologically stable disease. Eight patients with stable vision had a radiological progressive disease, while 3 of them had stable disease, 1 showed partial response, and 1 showed minor response at EOT.

**Figure 2:**
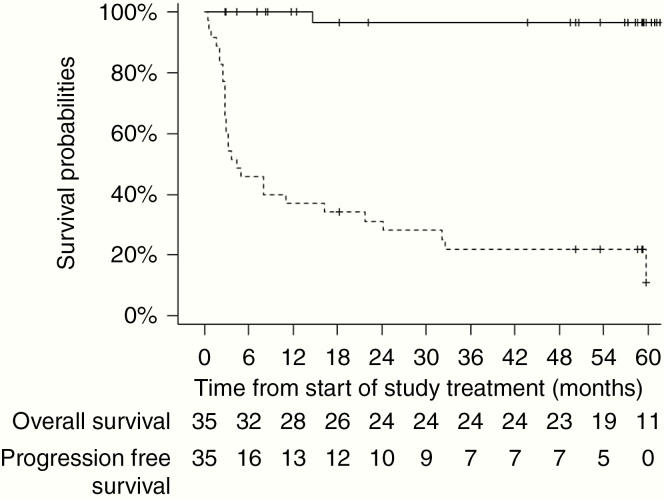
Kaplan–Meier curves of 5 years progression-free survival (dashed line) and 5 years overall survival (solid line) during the study for the 35 enrolled patients.

With a median follow-up of 58.3 months (range, 2.7–67.4), 28 patients experienced a disease progression and 1 patient died from disease progression of a chemo-refractory disease evolving for the last 14 years. The estimated 12-month PFS rate was 37.1% (95% CI, 23.2–53.67). PFS and OS curves are reported in Figure 2.

## Discussion

This phase I trial aimed at determining the recommended doses of vinblastine and nilotinib in combination for phase II trials (RP2D) using a dual-agent CRM model-based method. The identified RP2D for this population is 3 mg/m^2^ weekly for vinblastine and 230 mg/m^2^ BID for nilotinib.

The nilotinib dose and regimen are in line with previous studies in pediatric patients.^34,39^ A recent study on pharmacokinetic modeling of nilotinib monotherapy in patients 2–18 years old also confirmed this dose of 230 mg/m^2^.^40^ However, the vinblastine dose possible to combine with nilotinib was lower than those in the previously published study using 6 mg/m^2^ weekly injection regimen in monotherapy.^41^ The combination enhanced hematologic toxicity, which was the most frequent DLT in this study, followed by skin toxicities.

The main treatment-related adverse events in these children with LGG appeared consistent with those reported in patients with leukemia.^34,39^ Of note, no QT prolongation was observed in our population, which was reported in 25% in the leukemia cohort treated with nilotinib in monotherapy at the same dose and schedule.^34^ Among patients treated at the recommended dose, a majority (70%) has pursued the treatment without major dose reduction. Overall, the toxicity profile was acceptable and led to the phase II part of the VINILO study with the RP2D identified that randomizes the combination to vinblastine as monotherapy.

Regarding the enhanced incidence of hyperbilirubinemia, an association between *UGT1A1* polymorphisms and an increased risk of nilotinib-induced hyperbilirubinemia in adult patients with leukemia has been reported, but this was not described in a pediatric population.^42^ Results from the CAMN107A2101 trial in adult patients with imatinib-resistant/intolerant chronic myeloid leukemia or relapsed/refractory Philadelphia-positive acute lymphoblastic leukemia treated with nilotinib showed that patients with the (TA)7/(TA)7 genotype had a statistically significant higher risk of nilotinib-induced hyperbilirubinemia regardless of the dose of nilotinib.^42^ Those with the (TA)6/(TA)7 genotype had an increased risk of hyperbilirubinemia when compared to those with the normal genotype, but this difference did not reach statistical significance. We also observed this trend in our population, although the number of patients was too small to demonstrate statistical significance. Given the transient character of hyperbilirubinemia and that all patients recovered after treatment interruption, to be economically efficient, monitoring of bilirubin during initiation of nilotinib should be done, and Gilbert disease testing only if clinically suspected and other causes excluded. The need to reduce chemotherapy doses in this setting is debated.

Given the small number of dosages of nilotinib collected, we cannot firmly conclude about an association between toxicity and higher *C*_trough_. However, this association was reported in a previous study in adults with leukemia.^43^ The present case of grade 4 hypertension described in this study with the level of nilotinib still significant 4 days after the last dose is also coherent with these data. Trough concentrations in a limited number of patients were also concordant with previous studies, suggesting the absence of interaction of vinblastine on nilotinib plasmatic level.

The disease control rate of 85.3% (95% CI, 68.9–95.1) and the 12-month PFS of 37.1% (95% CI, 30–61) are below what is reported in the literature with vinblastine as a single agent in the setting of phase II. These results are however difficult to compare to published single-arm phase II studies with vinblastine as monotherapy due to different inclusion criteria and outcome measures.^41,44^ This question is evaluated in the randomized phase II part of the VINILO trial that is ongoing to compare both regimens.

For this phase I study, we evaluated, through a large simulation study, the operating characteristics of 2 dual-agent dose-escalation designs. The first design, developed by Yuan and Yin,^45^ converts the dual-agent dose escalation into sequential dose-escalation CRM designs, while the second one, developed by Wang and Ivanova,^35^ is an extension of the CRM design for a bidimensional space. As detailed in Supplementary Data II, this latter showed better operating characteristics compared to the first one in the VINILO setting, leading to the choice of this design for the current trial. We have developed a SAS program for this design (see Supplementary Data II indicating the link to have access to this program and 2 references).

In conclusion, this study shows that nilotinib can be safely combined with vinblastine, but at the expense of a 50% dose reduction of vinblastine. Radiological tumor responses and a high DCR could be observed in this study population despite the dose reduction of vinblastine and justified to proceed with the second part of the study. The latter randomizes the combination at the recommended doses (3 mg/m^2^ weekly of vinblastine combined with 230 mg/m^2^ BID of nilotinib) versus the standard treatment of weekly vinblastine alone in order to better explore the activity of the treatment combination.

## Supplementary Material

vdaa075_suppl_Supplementary_Material

## Notes

**Authorship statement**. Design and implementation of the trial: J.G., M.C.L.D., G.L.T., B.G., and G.V. Acquisition of the data: F.B., J.G., B.G., E.D.C., S.I.B., A.P., F.M., F.F., K.N., I.A., P.L., C.B., S.C., A.P.I., R.H.A., F.M.P., A.D.G., V.P., and D.V. Analysis and interpretation of the data: G.L.T., M.C.L.D., S.V., J.G., A.PI., M.E., V.P., and A.C. Manuscript writing: S.V., G.L.T., M.C.L.D., and J.G. All authors have been involved in the revision of the manuscript and have read and approved the final version.
