# Supplementary material for: Phase I study of vinblastine in combination with nilotinib in children, adolescents, and young adults with refractory or recurrent low-grade glioma
Source: Neurooncol Adv. 2020 Jun 9;2(1):vdaa075. doi: 10.1093/noajnl/vdaa075 (PMC7344116; doi:10.1093/noajnl/vdaa075)
Supplement: vdaa075_suppl_Supplementary_Material [file vdaa075_suppl_supplementary_material.docx]

## Supplementary Data

1. Toxicity definition and collection
2. Comparison of the operating characteristics of two dose-escalation Phase I designs for a dual agent in the VINILO Phase I trial setting (Tables S-1 to S-9)
3. Details of dose-escalation in the VINILO Phase I trial (Table S-10)
4. Adverse events, over the whole treatment duration, for the entire study population (Tables S-11, S-12)
5. Pharmacokinetic of nilotinib, related toxicity, and Gilbert disease association (Table S-13)

### Supplementary Data I: Toxicity definition and collection

Adverse events were evaluated on the whole treatment duration plus 28 days after end of treatment and graded according to the National Cancer Institute Common terminology Criteria (NCI-CTCAE version 4.0, <http://ctep.cancer.gov/protocolDevelopment/electronic_applications/ctc.htm>). Information about clinical adverse events (toxicity yes or no, grade if applicable, serious adverse event) was captured for each treatment cycle, using a list of 22 pre-defined toxicity terms corresponding to the expected adverse effects reported with the evaluated drugs ordered by system organ class (SOC):

- Blood and lymphatic system disorders: febrile neutropenia;
- Cardiac disorders: arrhythmia, palpitations, QT prolonged;
- Eye disorders: conjunctivitis, dry eye;
- Gastrointestinal disorders: abdominal pain, abdominal distension;
- Musculoskeletal disorders: bone pain, chest wall pain, myalgia;
- Nervous system disorders: dizziness, paresthesia;
- Respiratory disorders: dyspnea, epistaxis, cough, pleural effusion;
- Skin disorders: periorbital edema. Urticarial, erythema, dry skin, alopecia.

Additional adverse events not listed in the predefined list were collected using text fields, ordered by SOC.

Hematology and biochemistry tests were also captured in the database. Complete blood count (total white blood cell count, neutrophil and platelet counts, hemoglobin) was evaluated at baseline (within 7 days before study enrolment) and once a week during each cycle for the first 3 cycles, then at least before each cycle during study treatment period. Biochemistry tests (Sodium, potassium, phosphorus, magnesium, calcium, creatinine, AST/SGOT, ALT/SGPT, total bilirubin, albumin, urea, total protein, amylase and lipase) were performed at baseline (within 7 days before study enrolment), then before each cycle, and if possible, at the first post-discontinuation visit. In case of hypophosphatemia a full evaluation of phospho-calcic balance was necessary (Vitamin D, PTH levels). The frequency of the blood assessment could be adapted on the patient's clinical situation, but it was actually performed weekly for most patients. It was reported in the data base per 28-day cycles

### Supplementary Data II: Comparison of the operating characteristics of two dose-escalation Phase I designs for a dual agent in the VINILO Phase I trial setting

The VINILO phase I trial is a dual agent dose-finding oncology trial aiming to define the recommended phase 2 dose (RP2D) when combining nilotinib and vinblastine for a future phase II trial in refractory/recurrent low-grade glioma. Four dose levels of vinblastine could be explored (3, 4, 5 and 6 mg/m²/week), and three dose levels were planned for nilotinib (115, 230 and 350 mg/m^2^ BID). We did not plan to explore the combination of an increased dose of vinblastine with the lowest dose of nilotinib, i.e. (4;115), (5;115) and (6;115), (Table S-1). This choice was based on the rationale that a dose of 230 mg/m^2^ has been established in leukemia as the necessary dose to target the BCR-ABL. The affinity of the drug for this target is higher than the one for PDGFR α/β and other accessory targets. Moreover, to reach the tumor in the brain we thought that the higher doses possible of nilotinib would be desirable. Thus, nilotinib increase was prioritized in this protocol.

Also, the effective dose of vinblastine in this disease has not been clearly established and the current dose was derived from the one used in other diseases such as lymphoma. The 6 mg/m^2^/week dose is often reduced when used as maintenance: for example, it has been shown in anaplastic large cell lymphoma that the dose actually received during a one-year maintenance treatment as single agent was 4.7 mg/m2/week ^1^. We therefore considered that a lower starting dose of vinblastine may be acceptable and better applicable for a one-year period.

We evaluated two dual agent dose-escalation designs. The first one, published by Yuan and Yin ^2^, was chosen for its simplicity. This is a model-based design, derived from the continual reassessment method (CRM) ^3^ considering sequentially different subtrials with one fixed dose for one agent while escalating the dose of the second drug. One parameter is estimated for each subtrial to fit the dose-toxicity relationship between the dose of second drug and the probability of DLT, while the risk ratios between the different subtrials (i.e the different doses of the first drug) are prespecified and not estimated. The second design published by Wang and Ivanova ^4^ was chosen for its flexibility, as it allows the exploration of the bi-dimensional space considering all dose levels adjacent to the current dose level. This design is an extension of the CRM design ^3^ in a bi-dimensional space with a two-parameter dose-toxicity relationship modeling (one parameter for each agent).

In section 1, we describe briefly these two dual-agent dose-escalations Phase I designs. Section 2 presents the simulation study comparing the operating characteristics of both designs in the VINILO Phase I setting and section 3 reports the results.

**Table S-1: Dose combinations of vinblastine and nilotinib potentially explored in the VINILO phase I trial**

|  | **Vinblastine 3 mg/m2** | **Vinblastine 4 mg/m2** | **Vinblastine 5 mg/m2** | **Vinblastine 6 mg/m2** |
| --- | --- | --- | --- | --- |
| **Nilotinib 350 mg/m2** | • | • | • | • |
| **Nilotinib 230 mg/m2** | • | • | • | • |
| **Nilotinib 115 mg/m2** | • | Not explored | Not explored | Not explored |

1. **Dual-agent dose-escalation Phase I designs**

***Yuan and Yin approach***

The authors proposed to convert the dose-escalation design for dual agent into k one dimensional dose-escalation trials with k the number of dose levels of the second agent. When initiating the VINILO phase I trial, one main and two subtrials were considered (k=3). So, the bi-dimensional dose space was considered as three subtrials, with, for each subtrial, a dose-escalation of the first agent (vinblastine) for a fixed dose of second agent (nilotinib). The exploration of dose levels for the subtrials depends on the RP2D identified on the main subtrial. The main subtrial (dose level of nilotinib=230 mg/m2) is run firstly to find the RP2D. From this RP2D, we determine the search space of other subtrials by using it as a truncation boundary. Then, the low-dose (dose level of nilotinib=115 mg/m2) and high-dose (dose level of nilotinib=350 mg/m2) subtrials were run simultaneously. These 2 subtrials are not totally independent of the main trial since priors of low-dose and high-dose subtrials are proportional to the posterior toxicity probability of the main subtrial. We fixed the probability ratio at 1.15. For each subtrial, a Bayesian CRM with one-parameter empirical power model is used to assess the relation between the dose levels and the probability of DLT: $\phi\text{(}d,\alpha)=p{}_{0d}^{\exp(\alpha)}$ where $\phi\text{(}d,\alpha)$is the estimated probability of DLT at dose level d. *p_0d_* represents the initial guesses of toxicity probabilities DLT at dose level d (called also working model or skeleton), reflecting the clinicians’ prior belief and α is the unknown parameter to be estimated by the model. At the end of the trial, this method provides a recommended dose for each dose level of the second agent. See more details in Yuan et Yin ^2^. We used the R dfcrm package ^5^ with a non-informative Normal (0,1.34) prior distribution for α parameter in the Bayesian computation.

***Wang and Ivanova approach***

The authors proposed an approach based on an empirical model for a two-dimensional dose CRM finding design in a Bayesian framework. The probability of a dose limiting toxicity (DLT) is expressed as follows:

$$\varphi\left( s_{i},t_{j},\theta\right)=1-{(1-a_{i})}^{\alpha}{(1-b_{j})}^{\beta+\gamma log(1-a_{i})}$$

with $s_{i} (i=1,\ldots,I$) representing the dose levels of first agent and $t_{j} \left( j=1, \ldots, J \right)$ representing the dose levels of the second agent ($I>J)$. Here, the first agent is vinblastine with $I=4$ and the second agent is nilotinib with $J=3$. Although a start-up step, based on an algorithm for dose-escalation, may be proposed for this design in order to gather information not driven by the model, this will be not used in the setting of the VINILO trial. The vectors $a_{i} \left( i=1,\ldots,I \right)$ and $b_{j} \left( j=1,\ldots,J \right)$ represent the initial guesses of toxicity probabilities for the first and second agent (called also working model or skeleton), respectively. During the dose-escalation, the next cohort is treated at a dose combination where the estimated probability of toxicity is the closest to targeted toxicity probability. The authors recommend making the next dose assignment to a combination adjacent to the current one excluding the combination increasing the dose of both agents. At the end of the trial, the method provides the RP2D for each dose level of second agent. See more details in Wang et Ivanova ^4^. We developed an R function to implement this design with or without start-up. For SAS users, a SAS macro ^6,7^ is also available at <https://github.com/Oncostat/CRM2dim>. We considered no interaction between the 2 agents ($\gamma$ set to 0) and the prior distribution for the parameters $(\alpha, \beta)$ is the product of two independent exponentials with mean 1. The Bayesian estimation is based on 1000 Monte Carlo iterations.

1. **Simulation study**

We performed a simulation study to compare the operating characteristics of these two designs using different scenarios.

**2.1. Scenarios**

We repeated 1000 simulation runs of a dual-agent phase I trial for different scenarios (See Table S-2) with a number of dose levels for the first and second agent corresponding to those explored in the VINILO study. The scenarios 1-4 correspond to the first four scenarios used in the Wang’s paper. The scenarios 5-8 correspond to the first four scenarios used in the Yuan’s paper. The first and second agent corresponds to vinblastine and nilotinib, respectively.

**Table S-2: Probability of DLT for each dose combination in the different scenarios**

|  |  | **Vinblastine** | | | |
| --- | --- | --- | --- | --- | --- |
|  |  | dose 1 | dose 2 | dose 3 | dose 4 |
| **Nilotinib** | Scenario 1 | | | | |
|  | dose 3 | 0.08 | 0.13 | **0.20** | 0.29 |
|  | dose 2 | 0.05 | 0.08 | 0.13 | **0.20** |
|  | dose 1 | 0.03 | 0.05 | 0.08 | 0.13 |
|  | Scenario 2 | | | | |
|  | dose 3 | 0.05 | 0.08 | 0.11 | **0.15** |
|  | dose 2 | 0.04 | 0.06 | 0.09 | **0.13** |
|  | dose 1 | 0.04 | 0.05 | 0.08 | 0.11 |
|  | Scenario 3 | | | | |
|  | dose 3 | **0.20** | 0.30 | 0.41 | 0.53 |
|  | dose 2 | 0.10 | **0.20** | 0.25 | 0.32 |
|  | dose 1 | 0.03 | 0.05 | 0.13 | 0.20 |
|  | Scenario 4 | | | | |
|  | dose 3 | **0.20** | 0.40 | 0.47 | 0.56 |
|  | dose 2 | 0.08 | 0.13 | **0.20** | 0.32 |
|  | dose 1 | 0.03 | 0.05 | 0.08 | 0.13 |
|  | Scenario 5 | | | | |
|  | dose 3 | **0.20** | 0.30 | 0.40 | 0.50 |
|  | dose 2 | 0.10 | **0.20** | 0.30 | 0.40 |
|  | dose 1 | 0.05 | 0.12 | 0.20 | 0.30 |
|  | Scenario 6 | | | | |
|  | dose 3 | 0.10 | **0.20** | 0.30 | 0.40 |
|  | dose 2 | 0.05 | 0.12 | **0.20** | 0.30 |
|  | dose 1 | 0.04 | 0.06 | 0.08 | 0.12 |
|  | Scenario 7 | | | | |
|  | dose 3 | 0.30 | 0.42 | 0.52 | 0.62 |
|  | dose 2 | **0.20** | 0.30 | 0.40 | 0.50 |
|  | dose 1 | 0.02 | 0.06 | 0.12 | 0.20 |
|  | Scenario 8 | | | | |
|  | dose 3 | 0.11 | **0.21** | 0.31 | 0.42 |
|  | dose 2 | 0.10 | **0.20** | 0.30 | 0.40 |
|  | dose 1 | 0.08 | 0.19 | 0.29 | 0.39 |

Columns represent the dose level of first agent (Vinblastine). Rows represent the dose level of second agent (Nilotinib).

Maximum tolerated combinations (or combinations closest to them) for a target toxicity probability of 0.2 are in bold.

However, only some dose combinations were explored for dose-escalation for clinical interest purpose (Table S-1). As a consequence, a main trial including the combination (Nilotinib: 115, Vinblastine: 3) plus the different combinations for the dose level (Nilotinib: 230) and a high-dose subtrial trial (Nilotinib: 350) are considered when using the Yuan’s approach (See blue lines in Table S-1).

***2.2 Design parameters***

The sample size is set at 40 patients. The cohort size is equal to 2 as planned in the VINILO trial setting. For Yuan’s approach this sample size is divided into 24 patients in the main trial and 16 in the high subtrial. The trial stops when the pre-specified number of patients is reached. We did not allow skipping of dose levels during dose-escalation. No intra-patient dose-escalation is permitted. At least 2 patients fully observed with no DLT are requested at a given dose before dose-escalation. The definition of recommended dose is the dose level associated with an estimated DLT probability closest to the target toxicity probability set at 20% (target used for the VINILO phase I trial). We considered two sets of working models both for approaches (Table S-3) ^2,4^. For comparison, no start-up was used for the Wang’s approach.

**Table S-3: Prior probabilities (working models) and parameters used for the simulation**

|  | **Working models for Yuan approach** | **Working models for Wang approach** |
| --- | --- | --- |
| **Set 1** | p0d ={0.01, 0.05, 0.1, 0.2, 0.3} | {a1, ..., a4} = {0.05, 0.1, 0.2, 0.3}  {b1, ...,b3} = {0.05, 0.1, 0.2} |
| **Set 2** | p0d ={0.01, 0.02, 0.06, 0.12, 0.2} | {a1, ..., a4} = {0.02, 0.06, 0.12, 0.2}  {b1,...,b3} = {0.02, 0.06, 0.12} |

***2.3 Metrics***

The metrics to evaluate the operating characteristics of the dual-agent design included the percentage of correct selection (PCS) of the recommended dose (by dose level of second agent), the average number of patients treated at each dose level and the average number of observed DLT’s at each dose level.

1. **Results**

Tables S-4 to S-6 report the results when using the working model set 1 (See Table S-3). Table S-4 represents the distribution of the recommended dose. In 6 of 8 studied scenarios, the Wang’s method presented a PCS higher than that of Yuan’s approach. The control of overdosing is better with Wang’s method in 4 of 5 cases with a difference of PCS varying from 9.7 to 14.1%. Tables S-7 to S-9 report the results when using the working model set 2 of Table S-3.

**Table S-4: Percentage of correct selection by dose level of the second agent**

| **Scenario** | **Wang’s method (without start-up)** | | | | **Yuan’s method** | | | |
| --- | --- | --- | --- | --- | --- | --- | --- | --- |
|  | DL1 | DL2 | DL3 | DL4 | DL1 | DL2 | DL3 | DL4 |
| **Scenario 1** | 16.3 | 14.7 | **42.4** | 23.4 | 11.7 | 31.7 | **37.5** | 18.9 |
|  | 0.0 | 2.2 | 36.4 | **61.4** | 1.3 | 11.4 | 29.3 | **57.8** |
|  | 0.0 |  |  |  | 0.0 |  |  |  |
|  |  | | | |  | | | |
| **Scenario 2** | 2.6 | 2.9 | 18.5 | **75.0** | 3.6 | 16.1 | 31.9 | **48.2** |
|  | 0.0 | 0.2 | 9.0 | **90.8** | 1.3 | 7.8 | 17.8 | **72.9** |
|  | 0.0 |  |  |  | 0.0 |  |  |  |
|  |  | | | |  | | | |
| **Scenario 3** | **61.6** | 5.4 | 3.6 | 0.0 | **72.1** | 22.8 | 4.7 | 0.1 |
|  | 10.6 | **45.9** | 37.5 | 6.0 | 18.8 | **39.4** | 27.1 | 14.4 |
|  | 0.0 |  |  |  | 0.0 |  |  |  |
|  |  | | | |  | | | |
| **Scenario 4** | **79.4** | 4.2 | 1.8 | 0.1 | **79.0** | 18.2 | 2.6 | 0.0 |
|  | 2.4 | 30.3 | **58.4** | 8.9 | 6.6 | 29.3 | **41.5** | 22.4 |
|  | 0.0 |  |  |  | 0.0 |  |  |  |
|  |  | | | |  | | | |
| **Scenario 5** | **52.8** | 5.4 | 3.0 | 0.0 | **71.3** | 23.8 | 3.2 | 0.1 |
|  | 13.3 | **54.2** | 30.0 | 2.5 | 22.5 | **44.0** | 25.6 | 6.3 |
|  | 0.0 |  |  |  | 0.0 |  |  |  |
|  |  | | | |  | | | |
| **Scenario 6** | 47.3 | **17.7** | 19.6 | 2.8 | 27.5 | **48.0** | 21.9 | 2.3 |
|  | 1.3 | 18.8 | **59.3** | 20.6 | 4.5 | 27.5 | **40.5** | 27.2 |
|  | 0.0 |  |  |  | 0.0 |  |  |  |
|  |  |  |  |  |  |  |  |  |
| **Scenario 7** | 31.9 | 0.2 | 0.0 | 0.0 | 88.5 | 2.9 | 0.0 | 0.0 |
|  | **61.7** | 34.1 | 4.1 | 0.1 | **51.7** | 32 | 6.7 | 1.0 |
|  | 0.0 |  |  |  | 0.0 |  |  |  |
|  |  |  |  |  |  |  |  |  |
| **Scenario 8** | 41.8 | **9.0** | 8.3 | 0.2 | 45.9 | **40.4** | 9.0 | 0.2 |
|  | 11.8 | **49.1** | 32.8 | 6.3 | 22.5 | **43.4** | 23.5 | 6.1 |
|  | 0.0 |  |  |  | 0.0 |  |  |  |

Maximum tolerated combinations (or combinations closest to them) are in bold. Target toxicity probability = 20%. Working model for Wang’s approach: {a1,..., a4}={0.05, 0.1, 0.2, 0.3}, {b1, ...,b3}={0.05, 0.1, 0.2}. Working model for Yuan’s approach: *p_0d_*={0.01, 0.05, 0.1, 0.2, 0.3} and coefficient for high subtrial=1.15. n=40 patients

**Table S-5: Overall percentage of patients treated at each combination**

| **Scenario** | **Wang’s method (without start-up)** | | | | **Yuan’s method** | | | |
| --- | --- | --- | --- | --- | --- | --- | --- | --- |
|  | DL1 | DL2 | DL3 | DL4 | DL1 | DL2 | DL3 | DL4 |
| **Scenario 1** | 0.0 | 11.5 | **19.1** | 17.0 | 11.3 | 12.0 | **11.2** | 5.4 |
|  | 5.4 | 8.8 | 18.7 | **14.5** | 7.6 | 10.5 | 13.7 | **22.0** |
|  | 5.0 |  |  |  | 6.2 |  |  |  |
|  |  | | | |  | | | |
| **Scenario 2** | 0.0 | 5.8 | 12.0 | **43.0** | 8.1 | 9.9 | 10.6 | **11.4** |
|  | 5.2 | 6.3 | 10.5 | **12.2** | 7.3 | 8.9 | 10.5 | **26.7** |
|  | 5.0 |  |  |  | 6.6 |  |  |  |
|  |  | | | |  | | | |
| **Scenario 3** | **0.0** | 9.2 | 5.1 | 1.6 | **28.0** | 9.4 | 2.3 | 0.2 |
|  | 13.3 | **31.8** | 29.9 | 3.7 | 14.2 | **17.2** | 12.7 | 8.2 |
|  | 5.4 |  |  |  | 7.8 |  |  |  |
|  |  | | | |  | | | |
| **Scenario 4** | **0.0** | 9.9 | 4.5 | 1.7 | **28.7** | 9.0 | 2.1 | 0.1 |
|  | 8.0 | 25.8 | **39.9** | 5.0 | 10.3 | 14.7 | **16.2** | 12.1 |
|  | 5.1 |  |  |  | 6.7 |  |  |  |
|  |  | | | |  | | | |
| **Scenario 5** | **0.0** | 6.9 | 4.3 | 1.2 | **28.4** | 9.1 | 1.7 | 0.1 |
|  | 14.9 | **37.2** | 27.2 | 2.7 | 15.2 | **18.9** | 11.5 | 5.7 |
|  | 5.5 |  |  |  | 8.8 |  |  |  |
|  |  | | | |  | | | |
| **Scenario 6** | 0.0 | **13.3** | 12.9 | 4.7 | 16.5 | **14.9** | 7.4 | 1.1 |
|  | 6.6 | 19.0 | **30.9** | 7.5 | 8.9 | 13.9 | **16.6** | 13.6 |
|  | 5.0 |  |  |  | 7.0 |  |  |  |
|  |  |  |  |  |  |  |  |  |
| **Scenario 7** | 0.0 | 2.4 | 1.0 | 0.0 | 33.6 | 2.8 | 0.1 | 0.0 |
|  | **36.1** | 35.5 | 13.4 | 0.9 | **24.3** | 16.1 | 6.2 | 1.9 |
|  | 10.3 |  |  |  | 11.6 |  |  |  |
|  |  |  |  |  |  |  |  |  |
| **Scenario 8** | 0.0 | **7.9** | 6.8 | 1.9 | 21.1 | **13.6** | 3.4 | 0.2 |
|  | 14.7 | **34.6** | 25.2 | 3.3 | 14.9 | **18.1** | 10.9 | 5.3 |
|  | 5.6 |  |  |  | 10.8 |  |  |  |

Maximum tolerated combinations (or combinations closest to them) are in bold. Target toxicity probability = 20%. Working model for Wang’s approach: {a1, ..., a4}={0.05, 0.1, 0.2, 0.3}, {b1, ...,b3}={0.05, 0.1, 0.2}. Working model for Yuan’s approach: *p_0d_* ={0.01, 0.05, 0.1, 0.2, 0.3} and coefficient for high subtrial=1.15. n=40 patients

**Table S-6: Average number of toxicities observed at each combination**

| **Scenario** | **Wang’s method (without start-up)** | | | | **Yuan’s method** | | | |
| --- | --- | --- | --- | --- | --- | --- | --- | --- |
|  | DL1 | DL2 | DL3 | DL4 | DL1 | DL2 | DL3 | DL4 |
| **Scenario 1** | 0.0 | 1.5 | **3.7** | 4.9 | 1.6 | 1.6 | **1.6** | 1.6 |
|  | 0.3 | 0.7 | 2.5 | **2.9** | 0.4 | 0.8 | 1.8 | **4.4** |
|  | 0.2 |  |  |  | 0.2 |  |  |  |
|  |  | | | |  | | | |
| **Scenario 2** | 0.0 | 0.5 | 1.4 | **6.4** | 1.8 | 1.8 | 1.8 | **1.8** |
|  | 0.2 | 0.4 | 0.9 | **1.6** | 0.3 | 0.5 | 0.99 | **3.3** |
|  | 0.2 |  |  |  | 0.2 |  |  |  |
|  |  | | | |  | | | |
| **Scenario 3** | **0.0** | 2.8 | 2.2 | 0.9 | **0.1** | 0.1 | 0.1 | 0.1 |
|  | 1.3 | **6.4** | 7.3 | 1.2 | 1.4 | **3.4** | 3.2 | 2.6 |
|  | 0.2 |  |  |  | 0.3 |  |  |  |
|  |  | | | |  | | | |
| **Scenario 4** | 0. | 3.9 | 2.1 | 1.0 | **0.1** | 0.1 | 0.1 | 0.1 |
|  | 0.6 | 3.3 | 8.0 | 1.5 | 0.8 | 1.9 | **3.3** | 3.8 |
|  | 0.2 |  |  |  | 0.2 |  |  |  |
|  |  | | | |  | | | |
| **Scenario 5** | **0.0** | 2.1 | 1.7 | 0.6 | **0.1** | 0.1 | 0.1 | 0.1 |
|  | 1.5 | **7.4** | 7.9 | 1.1 | 1.5 | **3.7** | 3.5 | 2.2 |
|  | 0.3 |  |  |  | 0.4 |  |  |  |
|  |  | | | |  | | | |
| **Scenario 6** | 0.0 | **2.8** | 3.8 | 1.9 | 0.4 | **0.4** | 0.4 | 0.4 |
|  | 0.3 | 2.3 | **6.1** | 2.3 | 0.5 | 1.6 | **3.2** | 4.2 |
|  | 0.2 |  |  |  | 0.3 |  |  |  |
|  |  |  |  |  |  |  |  |  |
| **Scenario 7** | 0.0 | 1.1 | 0.5 | 0.2 | 0.1 | 0.0 | 0.0 | 0.0 |
|  | **7.2** | 10.4 | 5.3 | 0.4 | **4.9** | 4.7 | 2.5 | 0.8 |
|  | 0.2 |  |  |  | 0.2 |  |  |  |
|  |  |  |  |  |  |  |  |  |
| **Scenario 8** | 0.0 | 1.9 | 2.1 | 0.8 | 0.1 | **0.1** | 0.1 | 0.1 |
|  | 1.4 | 6.9 | 7.3 | 1.3 | 1.5 | **3.5** | 3.3 | 2.0 |
|  | 0.4 |  |  |  | 0.8 |  |  |  |

Maximum tolerated combinations (or combinations closest to them) are in bold. Target toxicity probability = 20%. Working model for Wang’s approach: {a1, ..., a4}={0.05, 0.1, 0.2, 0.3}, {b1, ...,b3}={0.05, 0.1, 0.2}. Working model for Yuan’s approach: *p_0d_* ={0.01, 0.05, 0.1, 0.2, 0.3} and coefficient for high subtrial=1.15. n=40 patients

**Table S-7: Percent of correct selection by dose level of the second agent**

| **Scenario** | **Wang’s method (without start-up)** | | | | **Yuan’s method** | | | |
| --- | --- | --- | --- | --- | --- | --- | --- | --- |
|  | DL1 | DL2 | DL3 | DL4 | DL1 | DL2 | DL3 | DL4 |
| **Scenario 1** | 8.8 | 20.6 | **44.9** | 24.7 | **12.1** | 30.4 | 35.9 | 21.5 |
|  | 0.0 | 2.5 | 35.2 | **62.3** | 0.7 | **9.2** | 27.6 | 62.4 |
|  | 0.0 |  |  |  | 0.0 |  |  |  |
|  |  | | | |  | | | |
| **Scenario 2** | 1.2 | 3.5 | 17.1 | **77.8** | 3.4 | 14.1 | 26.6 | 55.8 |
|  | 0 | 0.1 | 7.3 | **92.6** | 0.8 | 4.6 | 15.3 | 79.2 |
|  | 0.0 |  |  |  | 0.0 |  |  |  |
|  |  | | | |  | | | |
| **Scenario 3** | **61.4** | 14.9 | 5.0 | 0.2 | **71.7** | 22.9 | 4.4 | 0.2 |
|  | 5.5 | **54.0** | 35.0 | 5.5 | 15.3 | **39.3** | 27.8 | 16.8 |
|  | 0.0 |  |  |  | 0.0 |  |  |  |
|  |  | | | |  | | | |
| **Scenario 4** | **78.3** | 13.0 | 2.3 | 0.1 | **78.0** | 19.3 | 2.2 | 0.2 |
|  | 1.1 | 38.7 | **52.7** | 7.5 | 4.5 | 27.6 | **42.4** | 25.2 |
|  | 0.0 |  |  |  | 0.0 |  |  |  |
|  |  | | | |  | | | |
| **Scenario 5** | **58.2** | 14.3 | 3.4 | 0.1 | **71.2** | 23.4 | 3.3 | 0.1 |
|  | 6.2 | **64.5** | 26.7 | 2.6 | 17.9 | **47.0** | 26.0 | 7.1 |
|  | 0.0 |  |  |  | 0.0 |  |  |  |
|  |  | | | |  | | | |
| **Scenario 6** | 34.7 | **35.7** | 21.5 | 3.6 | 29.4 | **45.4** | 22.2 | 2.9 |
|  | 0.2 | 22.0 | **57.5** | 20.3 | 2.1 | 24.8 | **42.5** | 30.5 |
|  | 0.0 |  |  |  | 0.0 |  |  |  |
|  |  |  |  |  |  |  |  |  |
| **Scenario 7** | **45.2** | 1.4 | 0.0 | 0.0 | 86.1 | 3.5 | 0.1 | 0.0 |
|  | **50.3** | 45.9 | 3.8 | 0.0 | **46.2** | 34.8 | 7.7 | 1.0 |
|  | 0.0 |  |  |  | 0.0 |  |  |  |
|  |  |  |  |  |  |  |  |  |
| **Scenario 8** | 40.4 | **24.2** | 9.0 | 0.9 | 42.1 | **44.0** | 9.7 | 0.7 |
|  | 5.5 | **55.0** | 32.4 | 7.1 | 18.5 | **46.2** | 24.8 | 7.0 |
|  | 0.0 |  |  |  | 0.0 |  |  |  |

Maximum tolerated combinations (or combinations closest to them) are in bold. Target toxicity probability = 20%. Working model for Wang’s approach: {a1, …, a4}={0.02, 0.06, 0.12, 0.2}, {b1, …,b3}={0.02, 0.06, 0.12}. Working model for Yuan’s approach: *p_0d_*={0.01, 0.02, 0.06, 0.12, 0.2} and coefficient for high subtrial=(1.15), n=40 patients

**Table S-8: Overall percentage of patients treated at each combination**

| **Scenario** | **Wang’s method (without start-up)** | | | | **Yuan’s method** | | | |
| --- | --- | --- | --- | --- | --- | --- | --- | --- |
|  | DL1 | DL2 | DL3 | DL4 | DL1 | DL2 | DL3 | DL4 |
| **Scenario 1** | 0.0 | 9.7 | **18.7** | 20.5 | 11.4 | 11.7 | **10.9** | 5.9 |
|  | 5.1 | 8.6 | 18.2 | **14.3** | 6.8 | 10.0 | 12.9 | **24.1** |
|  | 5.0 |  |  |  | 6.1 |  |  |  |
|  |  | | | |  | | | |
| **Scenario 2** | 0.0 | 4.0 | 10.9 | **47.4** | 7.9 | 9.2 | 10.1 | **12.7** |
|  | 5.0 | 6.2 | 10.2 | **11.4** | 6.4 | 8.5 | 9.9 | **28.8** |
|  | 5.0 |  |  |  | 6.3 |  |  |  |
|  |  | | | |  | | | |
| **Scenario 3** | **0.0** | 11.1 | 6.5 | 1.9 | **27.7** | 9.6 | 2.2 | 0.3 |
|  | 8.9 | **37.3** | 25.4 | 3.8 | 12.7 | **17.6** | 12.3 | 9.8 |
|  | 5.0 |  |  |  | 7.7 |  |  |  |
|  |  | | | |  | | | |
| **Scenario 4** | **0.0** | 11.7 | 6.1 | 2.2 | **28.4** | 8.9 | 2.3 | 0.2 |
|  | 6.2 | 29.6 | **34.2** | 5.1 | 8.7 | 14.7 | **16.5** | 13.6 |
|  | 5.0 |  |  |  | 6.5 |  |  |  |
|  |  | | | |  | | | |
| **Scenario 5** | **0.0** | 10.4 | 5.2 | 1.5 | **27.2** | 10.2 | 1.7 | 0.1 |
|  | 9.4 | **42.9** | 22.6 | 2.9 | 13.0 | **19.4** | 12.5 | 6.7 |
|  | 5.0 |  |  |  | 8.4 |  |  |  |
|  |  | | | |  | | | |
| **Scenario 6** | 0.0 | **15.5** | 13.9 | 5.8 | 16.3 | **14.8** | 7.4 | 1.5 |
|  | 5.4 | 19.6 | **27.2** | 7.5 | 7.6 | 14.0 | **16.7** | 15.2 |
|  | 5.0 |  |  |  | 6.5 |  |  |  |
|  |  |  |  |  |  |  |  |  |
| **Scenario 7** | 0.0 | 4.0 | 1.4 | 0.4 | 32.9 | 2.7 | 0.2 | 0.0 |
|  | **29.9** | 45.8 | 12.0 | 1.0 | **21.6** | 16.6 | 6.8 | 2.2 |
|  | 5.5 |  |  |  | 12.9 |  |  |  |
|  |  |  |  |  |  |  |  |  |
| **Scenario 8** | 0.0 | **12.5** | 7.4 | 2.4 | 20.1 | **14.6** | 3.6 | 0.4 |
|  | 9.2 | **38.2** | 21.7 | 3.6 | 12.9 | **18.9** | 11.9 | 5.9 |
|  | 5.0 |  |  |  | 10.4 |  |  |  |

Maximum tolerated combinations (or combinations closest to them) are in bold. Target toxicity probability = 20%. Working model for Wang’s approach: {a1, …, a4}={0.02, 0.06, 0.12, 0.2}, {b1, …,b3}={0.02, 0.06, 0.12}. Working model for Yuan’s approach: *p_0d_*={0.01, 0.02, 0.06, 0.12, 0.2} and coefficient for high subtrial=(1.15), n=40 patients

**Table S-9: Average number of toxicities observed at each combination**

| **Scenario** | **Wang’s method (without start-up)** | | | | **Yuan’s method** | | | |
| --- | --- | --- | --- | --- | --- | --- | --- | --- |
|  | DL1 | DL2 | DL3 | DL4 | DL1 | DL2 | DL3 | DL4 |
| **Scenario 1** | 0.0 | 1.3 | **3.6** | 5.9 | 1.8 | 1.8 | **1.8** | 1.8 |
|  | 0.2 | 0.6 | 2.4 | **2.8** | 0.3 | 0.7 | 1.7 | **4.7** |
|  | 0.2 |  |  |  | 0.2 |  |  |  |
|  |  | | | |  | | | |
| **Scenario 2** | 0.0 | 0.3 | 1.2 | **7.0** | 1.9 | 1.9 | 1.9 | **1.9** |
|  | 0.2 | 0.3 | 0.9 | **1.5** | 0.3 | 0.5 | 0.9 | **3.6** |
|  | 0.2 |  |  |  | 0.3 |  |  |  |
|  |  | | | |  | | | |
| **Scenario 3** | **0.0** | 3.4 | 2.7 | 1.0 | **0.1** | 0.1 | 0.1 | 0.1 |
|  | 0.9 | **7.3** | 6.3 | 1.2 | 1.3 | **3.5** | 3.1 | 3.0 |
|  | 0.2 |  |  |  | 0.3 |  |  |  |
|  |  | | | |  | | | |
| **Scenario 4** | **0.0** | 4.7 | 2.9 | 1.3 | 0.1 | **0.1** | 0.1 | 0.1 |
|  | 0.5 | 3.8 | **6.8** | 1.5 | 0.7 | 1.8 | 3.3 | 4.4 |
|  | 0.2 |  |  |  | 0.2 |  |  |  |
|  |  | | | |  | | | |
| **Scenario 5** | **0.0** | 3.1 | 2.1 | 0.7 | **0.1** | 0.1 | 0.1 | 0.1 |
|  | 0.9 | **8.4** | 6.7 | 1.1 | 1.3 | **3.8** | 3.8 | 2.6 |
|  | 0.3 |  |  |  | 0.4 |  |  |  |
|  |  | | | |  | | | |
| **Scenario 6** | 0.0 | **3.2** | 4.2 | 2.3 | 0.6 | **0.6** | 0.6 | 0.6 |
|  | 0.3 | 2.3 | **5.4** | 2.3 | 0.4 | 1.5 | **3.3** | 4.5 |
|  | 0.2 |  |  |  | 0.3 |  |  |  |
|  |  |  |  |  |  |  |  |  |
| **Scenario 7** | 0.0 | 1.7 | 0.7 | 0.2 | 0.0 | 0.0 | 0.03 | 0.0 |
|  | **5.9** | 13.3 | 4.8 | 0.5 | **4.3** | 4.9 | 2.6 | 1.1 |
|  | 0.1 |  |  |  | 0.3 |  |  |  |
|  |  |  |  |  |  |  |  |  |
| **Scenario 8** | 0.00 | **2.6** | 2.3 | 0.9 | 0.2 | **0.2** | 0.2 | 0.1 |
|  | 0.9 | **7.5** | 6.4 | 1.4 | 1.2 | **3.7** | 3.6 | 2.3 |
|  | 0.4 |  |  |  | 0.9 |  |  |  |

Maximum tolerated combinations (or combinations closest to them) are in bold. Target toxicity probability = 20%. Working model for Wang’s approach: {a1, …, a4}={0.02, 0.06, 0.12, 0.2}, {b1, …,b3}={0.02, 0.06, 0.12}. Working model for Yuan’s approach: *p_0d_*={0.01, 0.02, 0.06, 0.12, 0.2} and coefficient for high subtrial=(1.15), n=40 patients

Sensitivity analysis: we also evaluated the operating characteristics (data not shown) with the following working model: $\left\{ a_{1}, a_{2}, a_{3}, a_{4} \right\}=\left\{ 0.07, 0.127, 0.200, 0.286 \right\}$ for vinblastine and $\left\{ b_{1},b_{2}, b_{3} \right\}=\left\{ 0.05, 0.1, 0.15 \right\}$ for nilotinib when using the Wang and Ivanova approach [3]. The working model for vinblastine was defined using the function getprior(0.04, 0.2, 3, 4, model=”empiric”) from dfcrm R package [4], ignoring the combination with nilotinib, and validated after discussion with the coordinating investigator. The working model for nilotinib was defined after discussion with the coordinating investigator, considering the expected probability of DLT for the whole set of dose combinations for $\alpha=1$ and $\beta=1$.

**Conclusion**

We performed a simulation study to compare the operating characteristics of the designs published by Yuan and Yin, and by Wang and Ivanova, two dose-escalation designs for drug combinations, through different scenarios and working models. It appeared that most of the times the design proposed by Wang and Ivanova showed better operating characteristics than the other one (higher percentage of correct selection of the recommended dose). Yuan et Yin design showed better characteristics only in a few scenarios corresponding to clinically unlikely scenarios; in these situations, the difference in favor of Yuan et Yin design was not substantial.

Based on these results, we thus considered that it was more appropriate to consider the statistical design proposed by Wang and Ivanova^4^ for the VINILO Phase I study.

**References**

1. Le Deley M-C, Rosolen A, Williams DM, et al. Vinblastine in children and adolescents with high-risk anaplastic large-cell lymphoma: results of the randomized ALCL99-vinblastine trial. *J Clin Oncol*. 2010;28(25):3987-3993.

2. Yuan Y, Yin G. Sequential continual reassessment method for two-dimensional dose finding. *Stat Med*. 2008;27(27):5664-5678.

3. O’Quigley J, Pepe M, Fisher L. Continual reassessment method: a practical design for phase 1 clinical trials in cancer. *Biometrics*. 1990;46(1):33-48.

4. Wang K, Ivanova A. Two-dimensional dose finding in discrete dose space. *Biometrics*. 2005;61(1):217-222.

5. Cheung, Y. K. *Dose Finding by the Continual Reassessment Method.* CRC Press; 2011.

6. Le Teuff G, Bayar M-A. *A Dose Escalation Method for Dual-Agent in Phase 1 Cancer Clinical Trial Using the SAS MCMC Procedure*.; 2016.

7. Bayar MA, Ivanova A, Le Teuff G. CRM2DIM: A SAS macro for implementing the dual-agent Bayesian continual reassessment method. *Comput Methods Programs Biomed*. 2019;176:211-223.

### Supplementary Data III: Details of dose-escalation in the VINILO Phase I trial

The following table describes the detail of the dose escalation process. For each new inclusion, we summarized the information available for previous recruited patients, at the date of dose allocation, leading to reassessment of the dose-toxicity relationship with update of the estimated probability of DLT at each dose level. Based on these probabilities, we could define the current recommended dose that should be allocated to the new patient. In some cases, the dose that was actually allocated differed from the dose recommended by the model, mainly due to safety decisions. They are detailed in the last column of the table below. The input parameters of the Wang and Ivanova approach [3] (see Supplementary Data II for the mathematical formulation of the model), when analyzing the data are: no interaction between the 2 agents ($\gamma=0$), the prior distribution of $\alpha$ and $\beta$ follow an exponential distribution of mean 1, iteration number is 5000 for Monte Carlo sampling, working model for vinblastine $\left\{ a_{1}, a_{2}, a_{3}, a_{4} \right\}=\left\{ 0.07, 0.127, 0.200, 0.286 \right\}$, working model for nilotinib $\left\{ b_{1},b_{2}, b_{3} \right\}=\left\{ 0.05, 0.1, 0.15 \right\}$ (see Supplementary Data II, sensitivity analysis for the specification of these priors).

**Table S-10: Definition of dose allocation for each new patient, based on prior observations**

|  |  |  |  | **Available information on  Number of DLT / Number of patients  at each dose level** | | | | **Estimated probability of DLT  at each dose level,  based on available information** | | | |  |  |  |
| --- | --- | --- | --- | --- | --- | --- | --- | --- | --- | --- | --- | --- | --- | --- |
| **Patient** | **Date of accrual** | **DLT** | **List of previous informative patients** | **(3;115)** | **(3;230)** | **(4;230)** | **(3;350)** | **(3;115)** | **(3;230)** | **(4;230)** | **(3;350)** | **Recom-mended dose based on the model^(1)^** | **Allocated dose ^(2)^** | **Comment if (1) and (2) differed** |
| prior |  |  |  | -/0 | -/0 | -/0 | -/0 | 0.11 | 0.16 | 0.20 | 0.20 |  |  |  |
| 1 | 12/07/13 | 0 | - | -/0 | -/0 | -/0 | -/0 | 0.11 | 0.16 | 0.20 | 0.20 |  | (3;115) | Starting dose |
| 2 | 17/07/13 | 0 | - | -/0 | -/0 | -/0 | -/0 | 0.11 | 0.16 | 0.20 | 0.20 |  | (3;115) | Starting dose |
| 3 | 13/09/13 | NE | #1, 2 | 0/2 | -/0 | -/0 | -/0 | 0.10 | 0.14 | 0.18 | 0.18 | (4;230) or (3;350) | (3;230) | No-skipping dose rule |
| 4 | 24/09/13 | NE | #1, 2 | 0/2 | -/0 | -/0 | -/0 | 0.10 | 0.14 | 0.18 | 0.18 | (4;230) or (3;350) | (3;230) | No-skipping dose rule |
| 5 | 30/09/13 | NE | #1, 2 | 0/2 | -/0 | -/0 | -/0 | 0.10 | 0.14 | 0.18 | 0.18 | (4;230) or (3;350) | (3;230) | No-skipping dose rule |
| 6 | 05/11/13 | 1 | #1, 2 | 0/2 | -/0 | -/0 | -/0 | 0.10 | 0.14 | 0.18 | 0.18 | (4;230) or (3;350) | (3;230) | No-skipping dose rule |
| 7 | 07/11/13 | 0 | #1, 2 | 0/2 | -/0 | -/0 | -/0 | 0.10 | 0.14 | 0.18 | 0.18 | (4;230) or (3;350) | (3;230) | No-skipping dose rule |
| 8 | 19/11/13 | 0 | #1, 2 | 0/2 | -/0 | -/0 | -/0 | 0.10 | 0.14 | 0.18 | 0.18 | (4;230) or (3;350) | (3;230) | No-skipping dose rule |
| 9 | 19/11/13 | 0 | #1, 2 | 0/2 | -/0 | -/0 | -/0 | 0.10 | 0.14 | 0.18 | 0.18 | (4;230) or (3;350) | (3;230) | No-skipping dose rule |
| 10 | 03/12/13 | NE | #1-6 | 0/2 | 1/1 | -/0 | -/0 | 0.14 | 0.20 | 0.25 | 0.26 | (3;230) | (3;115) | Decision for safety reason, waiting for information of #7- 9 (DLT in pt #6) |
| 11 | 06/12/13 | NE | #1-6 | 0/2 | 1/1 | -/0 | -/0 | 0.14 | 0.20 | 0.25 | 0.26 | (3;230) | (3;115) | Decision for safety reason, waiting for information of #7- 9 (DLT in pt #6) |
| 12 | 18/12/13 | 0 | #1-6 | 0/2 | 1/1 | -/0 | -/0 | 0.14 | 0.20 | 0.25 | 0.26 | (3;230) | (3;115) | Decision for safety reason, waiting for information of #7- 9 (DLT in pt #6) |
| 13 | 22/01/14 | 0 | #1-12 | 0/3 | 1/4 | -/0 | -/0 | 0.11 | 0.16 | 0.20 | 0.21 | (4;230) | (4;230) |  |
| 14 | 23/01/14 | 1 | #1-12 | 0/3 | 1/4 | -/0 | -/0 | 0.11 | 0.16 | 0.20 | 0.21 | (4;230) | (4;230) |  |
| 15 | 04/02/14 | 1 | #1-12 | 0/3 | 1/4 | -/0 | -/0 | 0.11 | 0.16 | 0.20 | 0.21 | (4;230) | (4;230) |  |
| 16 | 07/02/14 | 0 | #1-12 + 14 | 0/3 | 1/4 | 1/1 | -/0 | 0.14 | 0.20 | 0.26 | 0.25 | (3;230) | (3;230) |  |
| 17 | 11/02/14 | 0 | #1-12 + 14 | 0/3 | 1/4 | 1/1 | -/0 | 0.14 | 0.20 | 0.26 | 0.25 | (3;230) | (3;230) |  |
| 18 | 11/02/14 | 0 | #1-12 + 14 | 0/3 | 1/4 | 1/1 | -/0 | 0.14 | 0.20 | 0.26 | 0.25 | (3;230) | (3;230) |  |
| 19 | 12/03/14 | 0 | #1-18 | 0/3 | 1/7 | 2/3 | -/0 | 0.14 | 0.19 | 0.25 | 0.24 | (3;230) | (3;230) |  |
| 20 | 19/03/14 | 0 | #1-18 | 0/3 | 1/7 | 2/3 | -/0 | 0.14 | 0.19 | 0.25 | 0.24 | (3;230) | (3;230) |  |
| 21 | 17/04/14 | 0 | #1-19 | 0/3 | 1/8 | 2/3 | -/0 | 0.14 | 0.19 | 0.25 | 0.23 | (3;230) | (3;230) |  |
| 22 | 24/04/14 | 0 | #1-20 | 0/3 | 1/9 | 2/3 | -/0 | 0.13 | 0.18 | 0.24 | 0.22 | (3;230) | (3;230) |  |
| 23 | 29/04/14 | 0 | #1-20 | 0/3 | 1/9 | 2/3 | -/0 | 0.13 | 0.18 | 0.24 | 0.22 | (3;230) | (3;230) |  |
| 24 | 16/05/14 | 1 | #1-20 | 0/3 | 1/9 | 2/3 | -/0 | 0.13 | 0.18 | 0.24 | 0.22 | (3;230) | (3;230) |  |
| 25 | 12/06/14 | 0 | #1-23 | 0/3 | 1/12 | 2/3 | -/0 | 0.12 | 0.16 | 0.22 | 0.20 | (3;350) | (4;230) | Before amendment, dose level (3;350) not yet open |
| 26 | 20/06/14 | 0 | #1-24 | 0/3 | 2/13 | 2/3 | -/0 | 0.14 | 0.19 | 0.25 | 0.24 | (3;230) | (3;230) |  |
| 27 | 30/06/14 | 0 | #1-24 | 0/3 | 2/13 | 2/3 | -/0 | 0.14 | 0.19 | 0.25 | 0.24 | (3;230) | (3;230) |  |
| 28 | 02/12/14 | 0 | #1-27 | 0/3 | 2/15 | 2/4 | -/0 | 0.13 | 0.17 | 0.23 | 0.21 | (3;350) | (3;350) |  |
| 29 | 16/12/14 | 0 | #1-27 | 0/3 | 2/15 | 2/4 | -/0 | 0.13 | 0.17 | 0.23 | 0.21 | (3;350) | (3;350) |  |
| 30 | 18/12/14 | 0 | #1-27 | 0/3 | 2/15 | 2/4 | -/0 | 0.13 | 0.17 | 0.23 | 0.21 | (3;350) | (3;350) |  |
| 31 | 06/01/15 | 0 | #1-28 | 0/3 | 2/15 | 2/4 | 0/1 | 0.12 | 0.16 | 0.22 | 0.21 | (3;350) | (3;230) | Decision for safety reason,  waiting for information of #29-30;  already 3 patients at the dose level (3;350) |
| 32 | 04/02/15 | 1 | #1-30 | 0/3 | 2/15 | 2/4 | 0/3 | 0.11 | 0.15 | 0.21 | 0.19 | (4;230) | (4;230) |  |
| 33 | 20/05/15 | 0 | #1-32 | 0/3 | 2/16 | 3/5 | 0/3 | 0.13 | 0.17 | 0.23 | 0.21 | (3;350) | (3;350) |  |
| 34 | 20/05/15 | 1 | #1-32 | 0/3 | 2/16 | 3/5 | 0/3 | 0.13 | 0.17 | 0.23 | 0.21 | (3;350) | (3;350) |  |
| 35 | 09/07/15 | 0 | #1-34 | 0/3 | 2/16 | 3/5 | 1/5 | 0.14 | 0.18 | 0.25 | 0.22 | (3;230) | (3;230) |  |
| All |  |  | #1-35 | 0/3 | 2/17 | 3/5 | 1/5 | 0.13 | 0.18 | 0.24 | 0.22 | (3;230) |  |  |

**Supplementary Data IV: Adverse events, over the whole treatment duration, for the entire study population**

Table S-11: Maximum grade of adverse events reported over the whole treatment duration, considering all study patients (N=35, 246 cycles)

|  | Grade 1 | | Grade 2 | | Grade 3 | | Grade 4 | | Any grade | |
| --- | --- | --- | --- | --- | --- | --- | --- | --- | --- | --- |
| Type of Adverse event | **N** | **%** | **N** | **%** | **N** | **%** | **N** | **%** | **N** | **%** |
| Blood and lymphatic system disorders,  Any type | 7 | 20% | 9 | 26% | 12 | 34% | 6 | 17% | 34 | 97% |
| Anemia | 18 | 51% | 12 | 34% | 1 | 3% | 0 |  | 31 | 89% |
| Febrile neutropenia | 0 |  | 0 |  | 2 | 6% | 0 |  | 2 | 6% |
| Neutrophil count decreased | 1 | 3% | 9 | 26% | 11 | 31% | 6 | 17% | 27 | 77% |
| Platelet count decreased | 3 | 9% | 0 |  | 0 |  | 0 |  | 3 | 9% |
| White blood cell decreased | 16 | 46% | 8 | 23% | 3 | 9% | 2 | 6% | 29 | 83% |
| Cardiac disorders, Any type | 1 | 3% | 0 |  | 0 |  | 0 |  | 1 | 3% |
| Palpitations | 1 | 3% | 0 |  | 0 |  | 0 |  | 1 | 3% |
| Endocrine disorders, Any type | 1 | 3% | 1 | 3% | 0 |  | 0 |  | 2 | 6% |
| Cushingoid | 0 |  | 1 | 3% | 0 |  | 0 |  | 1 | 3% |
| Endocrine Other | 1 | 3% | 0 |  | 0 |  | 0 |  | 1 | 3% |
| Eye disorders, Any type | 6 | 17% | 1 | 3% | 0 |  | 0 |  | 7 | 20% |
| Blurred vision | 2 | 6% | 0 |  | 0 |  | 0 |  | 2 | 6% |
| Conjunctivitis | 4 | 11% | 1 | 3% | 0 |  | 0 |  | 5 | 14% |
| Dry eye | 1 | 3% | 0 |  | 0 |  | 0 |  | 1 | 3% |
| Eye pain | 1 | 3% | 0 |  | 0 |  | 0 |  | 1 | 3% |
| Gastrointestinal disorders, Any type | 20 | 57% | 9 | 26% | 1 | 3% | 0 |  | 30 | 86% |
| Abdominal pain | 15 | 43% | 4 | 11% | 0 |  | 0 |  | 19 | 54% |
| Constipation | 7 | 20% | 1 | 3% | 0 |  | 0 |  | 8 | 23% |
| Diarrhea | 7 | 20% | 1 | 3% | 0 |  | 0 |  | 8 | 23% |
| Dyspepsia | 1 | 3% | 0 |  | 0 |  | 0 |  | 1 | 3% |
| Mucositis oral | 2 | 6% | 0 |  | 0 |  | 0 |  | 2 | 6% |
| Nausea | 12 | 34% | 1 | 3% | 0 |  | 0 |  | 13 | 37% |
| Vomiting | 14 | 40% | 5 | 14% | 1 | 3% | 0 |  | 20 | 57% |
| Gastrointestinal, Other | 1 | 3% | 0 |  | 0 |  | 0 |  | 1 | 3% |
| General disorders, Any type | 16 | 46% | 9 | 26% | 1 | 3% | 0 |  | 26 | 74% |
| Anorexia | 13 | 37% | 3 | 9% | 0 |  | 0 |  | 16 | 46% |
| Asthenia | 15 | 43% | 8 | 23% | 0 |  | 0 |  | 23 | 66% |
| Fever | 9 | 26% | 2 | 6% | 1 | 3% | 0 |  | 12 | 34% |
| Irritability | 1 | 3% | 0 |  | 0 |  | 0 |  | 1 | 3% |
| Weight loss | 1 | 3% | 1 | 3% | 0 |  | 0 |  | 2 | 6% |
| Infections and infestations, Any type | 9 | 26% | 5 | 14% | 1 | 3% | 0 |  | 15 | 43% |
| Bronchial infection | 1 | 3% | 2 | 6% | 0 |  | 0 |  | 3 | 9% |
| Lung infection | 0 |  | 1 | 3% | 0 |  | 0 |  | 1 | 3% |
| Skin infection | 4 | 11% | 1 | 3% | 0 |  | 0 |  | 5 | 14% |
| Upper respiratory infection | 6 | 17% | 4 | 11% | 1 | 3% | 0 |  | 11 | 31% |
| Investigations, Any type | 12 | 34% | 13 | 37% | 9 | 26% | 1 | 3% | 35 | 100% |
| Alanine aminotransferase increased | 22 | 63% | 6 | 17% | 5 | 14% | 0 |  | 33 | 94% |
| Alkaline phosphatase increased | 1 | 3% | 0 |  | 0 |  | 0 |  | 1 | 3% |
| Aspartate aminotransferase increased | 23 | 66% | 3 | 9% | 2 | 6% | 0 |  | 28 | 80% |
| Blood bilirubin increased | 13 | 37% | 7 | 20% | 1 | 3% | 0 |  | 21 | 60% |
| Creatinine increased | 2 | 6% | 0 |  | 0 |  | 0 |  | 2 | 6% |
| Hypercalcemia | 3 | 9% | 0 |  | 0 |  | 0 |  | 3 | 9% |
| Hyperkalemia | 3 | 9% | 1 | 3% | 1 | 3% | 0 |  | 5 | 14% |
| Hypermagnesemia | 4 | 11% | 0 |  | 0 |  | 0 |  | 4 | 11% |
| Hypernatremia | 5 | 14% | 0 |  | 0 |  | 0 |  | 5 | 14% |
| Hypoalbuminemia | 6 | 17% | 0 |  | 0 |  | 0 |  | 6 | 17% |
| Hypocalcemia | 19 | 54% | 0 |  | 1 | 3% | 0 |  | 20 | 57% |
| Hypokalemia | 20 | 57% | 0 |  | 1 | 3% | 0 |  | 21 | 60% |
| Hypomagnesemia | 18 | 51% | 2 | 6% | 0 |  | 0 |  | 20 | 57% |
| Hyponatremia | 18 | 51% | 0 |  | 1 | 3% | 1 | 3% | 20 | 57% |
| Hypophosphatemia | 10 | 29% | 1 | 3% | 0 |  | 0 |  | 11 | 31% |
| Lipase increased | 1 | 3% | 1 | 3% | 1 | 3% | 0 |  | 3 | 9% |
| Serum amylase increased | 1 | 3% | 0 |  | 0 |  | 0 |  | 1 | 3% |
| Musculoskeletal and connective tissue disorders, Any type | 6 | 17% | 1 | 3% | 0 |  | 0 |  | 7 | 20% |
| Back pain | 4 | 11% | 0 |  | 0 |  | 0 |  | 4 | 11% |
| Bone pain | 1 | 3% | 1 | 3% | 0 |  | 0 |  | 2 | 6% |
| Chest wall pain | 2 | 6% | 0 |  | 0 |  | 0 |  | 2 | 6% |
| Nervous system disorders, Any type | 15 | 43% | 12 | 34% | 1 | 3% | 0 |  | 28 | 80% |
| Concentration impairement | 1 | 3% | 0 |  | 0 |  | 0 |  | 1 | 3% |
| Dizziness | 2 | 6% | 0 |  | 0 |  | 0 |  | 2 | 6% |
| Gait disturbance | 1 | 3% | 0 |  | 0 |  | 0 |  | 1 | 3% |
| Headache | 12 | 34% | 9 | 26% | 0 |  | 0 |  | 21 | 60% |
| Hypersomnia | 1 | 3% | 0 |  | 0 |  | 0 |  | 1 | 3% |
| Nystagmus | 1 | 3% | 0 |  | 0 |  | 0 |  | 1 | 3% |
| Peripheral motor neuropathy | 1 | 3% | 1 | 3% | 0 |  | 0 |  | 2 | 6% |
| Peripheral sensory neuropathy | 14 | 40% | 2 | 6% | 0 |  | 0 |  | 16 | 46% |
| Seizure | 1 | 3% | 2 | 6% | 1 | 3% | 0 |  | 4 | 11% |
| Tremor | 1 | 3% | 0 |  | 0 |  | 0 |  | 1 | 3% |
| Vertigo | 1 | 3% | 0 |  | 0 |  | 0 |  | 1 | 3% |
| Nervous, Other | 1 | 3% | 0 |  | 0 |  | 0 |  | 1 | 3% |
| Psychiatric disorders, Any type | 4 | 11% | 4 | 11% | 0 |  | 0 |  | 8 | 23% |
| Agitation | 1 | 3% | 2 | 6% | 0 |  | 0 |  | 3 | 9% |
| Anxiety | 2 | 6% | 0 |  | 0 |  | 0 |  | 2 | 6% |
| Insomnia | 1 | 3% | 1 | 3% | 0 |  | 0 |  | 2 | 6% |
| Personality change | 1 | 3% | 2 | 6% | 0 |  | 0 |  | 3 | 9% |
| Renal and urinary disorders, Any type | 1 | 3% | 0 |  | 0 |  | 0 |  | 1 | 3% |
| Urinary tract pain | 1 | 3% | 0 |  | 0 |  | 0 |  | 1 | 3% |
| Respiratory, thoracic and mediastinal disorders, Any type | 11 | 31% | 1 | 3% | 0 |  | 0 |  | 12 | 34% |
| Cough | 10 | 29% | 0 |  | 0 |  | 0 |  | 10 | 29% |
| Dyspnea | 0 |  | 1 | 3% | 0 |  | 0 |  | 1 | 3% |
| Epistaxis | 1 | 3% | 0 |  | 0 |  | 0 |  | 1 | 3% |
| Skin and subcutaneous tissue disorders , Any type | 16 | 46% | 12 | 34% | 1 | 3% | 0 |  | 29 | 83% |
| Alopecia | 5 | 14% | 1 | 3% | 0 |  | 0 |  | 6 | 17% |
| Bullous dermatitis | 0 |  | 1 | 3% | 0 |  | 0 |  | 1 | 3% |
| Dry skin | 10 | 29% | 0 |  | 0 |  | 0 |  | 10 | 29% |
| Periorbital edema | 2 | 6% | 0 |  | 0 |  | 0 |  | 2 | 6% |
| Pruritus | 4 | 11% | 4 | 11% | 0 |  | 0 |  | 8 | 23% |
| Rash | 16 | 46% | 9 | 26% | 1 | 3% | 0 |  | 26 | 74% |
| Skin hyperpigmentation | 1 | 3% | 0 |  | 0 |  | 0 |  | 1 | 3% |
| Urticaria | 3 | 9% | 2 | 6% | 0 |  | 0 |  | 5 | 14% |
| Skin Other | 4 | 11% | 0 |  | 0 |  | 0 |  | 4 | 11% |
| Vascular disorders, Any type | 1 | 3% | 1 | 3% | 0 |  | 1 | 3% | 3 | 9% |
| Hematoma | 1 | 3% | 1 | 3% | 0 |  | 0 |  | 2 | 6% |
| Hypertension | 0 |  | 0 |  | 0 |  | 1 | 3% | 1 | 3% |
| Thromboembolic event | 0 |  | 1 | 3% | 0 |  | 0 |  | 1 | 3% |
| Other, Any type | 0 |  | 1 | 3% | 0 |  | 0 |  | 1 | 3% |
| Intra Tumoral Bleeding | 0 |  | 1 | 3% | 0 |  | 0 |  | 1 | 3% |

The adverse event terms have been coded according to MedDRA classification, by System Organ Class (SOC). In a clinical perspective, we have considered all hematological AEs in the SOC “Blood and lymphatic system disorders”. We have also re-classified “Anorexia” and “Weight loss” in the SOC “General disorder”. Lastly, we have pooled in the SOC “Investigation” all biological AEs other than hematological AE.

For each type of adverse event, we considered the maximum grade over the whole treatment duration. We only excluded adverse events unequivocally related to the disease.

The percentages equal to 0 have been removed from the table to facilitate the reading.

Table S-12: Number of cycles with neutropenia or febrile neutropenia per patient

|  | (3;115) N=5 | | (3;230) N=20 | | (4;230) N=5 | | | (3;350) N=5 | | | Overall N=35 | | |
| --- | --- | --- | --- | --- | --- | --- | --- | --- | --- | --- | --- | --- | --- |
|  | **N** | **%** | **N** | **%** | **N** | **%** | **N** | | **%** | **N** | | **%** |
| Number of cycles with  grade 3 or 4 neutropenia (febrile or not) |  |  |  |  |  |  |  | |  |  | |  |
| 0 | 2 | 40% | 13 | 65% | 1 | 20% | 2 | | 40% | 18 | | 51% |
| 1 | 1 | 20% | 1 | 5% | 1 | 20% | 2 | | 40% | 5 | | 14% |
| 2 | 0 |  | 3 | 15% | 2 | 40% | 1 | | 20% | 6 | | 17% |
| 3 or more | 2 | 40% | 3 | 15% | 1 | 20% | 0 | |  | 6 | | 17% |
| Number of cycles with  grade 3 or 4 febrile neutropenia |  |  |  |  |  |  |  | |  |  | |  |
| 0 | 5 | 100% | 20 | 100% | 4 | 80% | 4 | | 80% | 33 | | 94% |
| 1 | 0 |  | 0 |  | 1 | 20% | 0 | |  | 1 | | 3% |
| 3 | 0 |  | 0 |  | 0 |  | 1 | | 20% | 1 | | 3% |

### Supplementary Data V: Pharmacokinetic of Nilotinib, related toxicity, and Gilbert disease association

Table S-13: Pharmacokinetic data of nilotinib with trough concentration (C_trough_), toxicity, and Gilbert disease (n=11)

| **Patient number** | **Nilotinib dose** | **Day and Cycle of drug dosage** | **Timing of dosage** | **[NILO] ng/mL** | **Mean**  **ng/mL** | **Toxicity (Grade)** | **Gilbert disease** |
| --- | --- | --- | --- | --- | --- | --- | --- |
| **1** | 70% of 230 mg/m^2^/d | D1C10 | C_trough_ | *366* |  | Fatigue (G1), Cutaneous (G1) | heterozygote |
| **6** | 33% of 460 mg/m^2^/d | D8C4 | C_trough_ | 798 | 923.67 | Dermatologic (G1) at dosages,  previously dermatologic (G3) and reason for dose reduction | No |
|  |  | D15C5 | C_trough_ | 1065 |  |  |  |
|  |  | D15C11 | C_trough_ | 908 |  |  |  |
| **14** | 100% of 460 mg/m^2^/d | D22C8 | NA | 1117 |  | Dermatologic (G1) | heterozygote |
| **15** | 100% of 460 mg/m^2^/d | D22C8 | C_trough_ | 1523 |  | Fatigue (G1), hyperbilirubinemia (G1) at dosage,  Neutropenia (G3) during C1 | heterozygote |
| **18** | 100% of 460 mg/m^2^/d | D22C8 | C_trough_ | *2064* |  | No | heterozygote |
| **22** | 100% of 460 mg/m^2^/d | D1C6 | NA | 539 |  | No | NA |
| **30** | 100% of 700 mg/m^2^/d | D15C4 | NA | 815.5 | 1066.4 | Dermatologic (G1) | NA |
|  |  | D1C7 | 6h post | 1317.3 |  | No |  |
| **32** | 100% of 460 mg/m^2^/d | D18C1 | 4 days later | *123* |  | Hypertension (G4),  febrile neutropenia (G3) | NA |
|  | stop |  | 11 days later | *6.2* |  |  |  |
|  | stop |  | 24 days later | *<5* |  |  |  |
| **33** | 100% of 700 mg/m2/d | D8C1 | NA | 1418.2 | 1036.6 | No | NA |
|  |  | D1C3 | C_trough_ | 655 |  | Paresthesia (G1), Cutaneous (G2) | NA |
|  | stop | D1C4 | 14 days later | *9.2* |  | Dermatologic (G2) D15C3, Hepatic (G3) D21C3 |  |
|  | 50% of 700 mg/m^2^/d | D22C4 | C_trough_ | *1899* |  | Dermatologic (G1), hepatic (G1) |  |
| **34** | 100% of 700 mg/m^2^/d | D25C1 | NA | *3390.3* |  | ALT and AST (G3) | NA |
|  | 50% of 700 mg/m^2^/d | D15C2 | C_trough_ | *2752* |  | Previous dose reduction for dermatologic (G2) |  |
| **35** | 100% of 460 mg/m^2^/d | D8C1 | C_trough_ | 895 |  | NA | NA |

NA: not available
